# A fast numerical integration scheme for clonal expansion processes on graphs

**DOI:** 10.1371/journal.pcbi.1012784

**Published:** 2026-08-04

**Authors:** Chay Paterson, Miaomiao Gao, Joshua Hellier, Georg Luebeck, David C. Wedge, Ivana Bozic

**Affiliations:** 1 University of Manchester, Manchester, United Kingdom of Great Britain and Northern Ireland; 2 Fred Hutchinson Cancer Center, Seattle, United States of America; 3 University of Washington, Seattle, United States of America; University of Washington, UNITED STATES OF AMERICA

## Abstract

Compound birth-death processes are widely used to model the age-incidence curves of many cancers. There are efficient schemes for directly computing the relevant probability distributions in the context of linear multi-stage clonal expansion (MSCE) models. However, these schemes have not been generalised to models on arbitrary graphs, forcing the use of either full stochastic simulations or mean-field approximations, which can become inaccurate at late times or old ages. Here, we present a numerical integration scheme for directly computing survival probabilities of a first-order birth-death process on an arbitrary directed graph, without the use of stochastic simulations. As a concrete application, we show that this new numerical method can be used to infer the parameters of an example graphical model from simulated data.

## Author summary

In this work, we develop an algorithm that solves a first-order stochastic multi-stage model faster than existing methods. The main application is modelling cancer incidence as a function of age, but many other applications are imaginable. In this class of models, cancer cells are produced from a large number of healthy cells by a series of a few rare and random mutations. Multi-stage models have been very successful, and are currently widely used to explain why cancer is more common in old age. However, these multi-stage models only consider the overall number of stages, and do not consider different types of mutation, which could occur in different orders. The different orders in which different types of mutation can happen form a network or “graph”. This is because there was not an efficient algorithm for working out the age-specific incidence for multi-stage models on graphs. We have now found an algorithm for accurately computing this, at least ten million times faster than other proposals. We also show how this algorithm could be used to fit models to data from a simulated clinical study.

## 1 Introduction

The relationship between age and cancer incidence has provided crucial biological insights since it was first noted by Armitage and Doll [1–3]. It was recognised that the incidence could be modelled as the hazard function of a compound random process, with successive steps corresponding to accumulating mutations in a population of pre-neoplastic cells [1,4]. Today, multi-stage clonal expansion (MSCE) models are an established paradigm for studies of cancer, age, and public health: they have been instrumental in the discovery of tumour suppressor genes, such as Rb1; studies of environmental risk factors, such as radon exposure; and have enabled inferences about the number of “hits” involved in the initiation of a given neoplasm [3,5–8].

Existing MSCE models do not model alterations on specific loci, nor do they distinguish between different mutational mechanisms: for example, either a nonsense mutation or a large deletion could both result in loss-of-function of a tumour suppressor gene. These models consider only the overall number of hits, so that the successive stages are represented by nodes on a path graph [5,8,9]. This was an acceptable abstraction when age structure was the only variable of interest [6]. To extend the theory to include data about what mutational mechanisms have affected specific loci, we consider the stages as nodes on a general directed graph. This allows models to distinguish between different mutational mechanisms, such as point mutations and copy number alterations [10,11]. Our goal in this work is to describe a fast and arbitrarily accurate scheme for estimating age-structured incidence in these general MSCE models.

For all MSCE models, the key outputs are the survival probabilities $S_{f}(t)$ and hazard functions $h_{f}(t)$ for each subtype *f*, from which age-specific incidence can be estimated (see appendix A). As stochastic processes, $S_{f}(t)$ in these models can be computed in a natural way by sampling many replicates of stochastic simulations generated by e.g. Gillespie’s algorithm. However, such stochastic simulations are computationally expensive. Faster, semi-analytical methods to directly estimate $S_{f}(t)$ and $h_{f}(t)$ numerically, given some set of model parameters, are therefore highly desirable for statistical fitting. This was the goal of our work presented here.

MSCE models on path graphs have very well-studied methods for reducing the computation of $S_{f}(t)$ to numerical quadrature [5,6,8,12,13]. These methods study $S_{f}(t)$ via an intermediate generating function, conventionally denoted Ψ or Φ, and use Kolmogorov backward equations to derive recursive formulae for the probabilities $S_{f}(t)$ [4,12]. The probabilities $S_{f}(t)$ may then be evaluated using an numerical integration scheme such as Gaussian quadrature [5]. Kolmogorov *forward* equations have also been studied in this context, although they have not been explored as intensively [8,14], and interest in this approach waned.

No general approach for reducing the computation of $S_{f}(t)$ to quadrature on arbitrary directed graphs is known, although we are also aware of recent proposals by Tang et al. that extend the above classical methods [15]. The mutational transitions studied in MSCE models of cancer incidence have therefore been mainly limited to path graphs; and the kinetics are first order in the precursor cell populations [5,16,17]. More general models involving birth-death-mutation processes on arbitrary directed graphs have been studied using stochastic simulations, or approximate solutions derived from mean-field or moment closure approximations [10,18,19]. A limited number of analytical results for first-order chemical reaction networks are also known. Notably, monomolecular systems in which there is no more than one product per reaction, are known to be exactly solvable, although this is too restrictive to apply to MSCE models [20–24].

Due to the importance of stochasticity in biological systems, the discovery of exact analytical, semi-analytical, and numerical solutions is still an important open problem, given that stochastic simulation is computationally expensive [21,25,26].

Here, we describe an improved numerical scheme based on Kolmogorov forward equations and the method of characteristics [12,14]. We show how when the parameters of a clonal expansion models are constant, the resulting symmetries allow a massive reduction of the complexity, and the problem of finding survival probabilities can be reduced to integration of a small system of ordinary differential equations, even for arbitrary directed graphs. Not only is this new method much faster than stochastic sampling, it is *asymptotically* faster, meaning that the improvement in speed accelerates as the desired accuracy becomes finer.

## 2 Results: A new algorithm for estimating $S_{f}(t)$

The problem being considered is the estimation of the diagnosis-free survival curve $S_{f}(t)$ for a clonal expansion model on a graph *G*. The graph *G* consists of a set of vertices *V*, interpreted as different mutant genotypes in an intermediate metaplasia; and a set of edges *E*, interpreted as different mechanisms by which driver mutations can be acquired.

Different vertices in *V* correspond to subpopulations with different combinations of genetic alterations. Each subpopulation $N_{j}$, j∈V, is a random variable that is a non-negative integer, so that $N_{j}\in {\mathbb{Z}}_{\geq 0}$. The initial value of $N_{j}$ at age *t* = 0 will be denoted $Z_{j}$.

There is also a subset of nodes F∈V which are the “final” subpopulations. These are interpreted as neoplastic or cancerous cells. Our overarching goal is to compute the probability $S_{f}(t)$ that an individual has survived to age *t* with no primary tumours of type *f*. This is related to the probability *t*hat a chosen final node f∈F is unoccupied by time *t*: $P(t,N_{f}=0)$ (see Appendix A in S1 File). In existing MSCE models, the different cell types *j* and *k* represent nodes on a path graph with only one final node *f* [5,8,9].

The subpopulations $N_{j}$ change by four types of Poisson process, with the following “chemical reactions” and rates:

- Mutation (by asymmetric division): j→j+k, at rate $\mu_{jk}N_{j}$
- Birth (by symmetric division): j→j+j, at rate $\alpha_{j}N_{j}$
- Death: j→∅, at rate $\beta_{j}N_{j}$
- Immigration (to approximate mutations appearing in a large unobservable population [5]): ∅→j, at rate $\kappa_{j}$

In general, the rate coefficients $\left\{\alpha_{j},\beta_{j},\mu_{jk},\kappa_{j}\right\}$ may vary with respect to age or time: however, for the time being we will only consider the special case in which they are constant, as some dramatic optimisations are then possible.

The edge set *E* corresponds to pairs of nodes for which the mutation rate $\mu_{jk}>0$, and are interpreted as different mutational mechanisms. These may be mutations on different loci, or different biological processes such as deletions, loss of heterozygosity, copy number alterations and so on. For example, the graph may be a tree, with different vertices representing different ancestral genotypes. There are many imaginable applications: a directed graph of this type can encode any string [27].

The set of nodes *V* and edge set *E* together define a graph *G*=(*V*,*E*) [28]. In contrast to Luebeck and Moolgavkar, we will not assume a specific linear structure for this graph, and will instead study methods suitable for arbitrary graphs *G* [8].

These dynamics imply the following form for the Kolmogorov forward equations:


dP(t,N0,N1,...)dt=∑j∈V(αj(Nj−1)P(t,N0,...,Nj−1,...)+βj(Nj+1)P(t,N0,...,Nj+1,...)∑ab+∑k∈VμkjNkP(t,N0,...,Nk,...,Nj−1,...)+κjP(t,N0,...,Nj−1,...)−(αjNj+βjNj+∑k∈VμjkNj+κj)P(t,N0,...,Nj,...)∑ab) (1)

The Kolmogorov forward equations are also known as the chemical master equation [25,29,30]. In general, this system of ordinary differential equations is infinite-dimensional, and known solution methods are extremely limited [21]. The only general method for computing arbitrarily precise solutions for the distribution $P(t,\vec{N})$ is Gillespie’s stochastic simulation algorithm [31].

### 2.1 Intuition

Because the discussion of the details of our numerical algorithms is abstract, and not every term has a clear biological interpretation, we will describe in non-technical terms an intuitive explanation for our overall goal, and why our approach should work. To estimate the survival curve $S_{f}(t)$ (and more generally $P(t,\vec{N})$) for any given model, we must choose how to compute it using one of two types of approach: either by sampling many Monte Carlo simulations, or by finding a way to directly construct the probability distribution $P(t,\vec{N})$ that solves (1). The first of these is simple to implement and understand, but the empirical probabilities converge very slowly. The second is difficult, since there are a potentially unlimited number of relevant terms in (1), and these all have to be solved for simultaneously at each time step: usually one must exploit some special mathematical property or symmetry [26]. Our approach is of the second type: by changing our representation of the problem, we can solve a very small system of equations instead, which can also be solved sequentially instead of simultaneously.

There is also a deeper reason *why* changing the representation of this system should result in a dramatic simplification. System (1) has a key property: all the reactions on the right-hand side connect states $\vec{N}$ which are very “close” to one another, so that each of the populations in the neighbouring state are only different by ±1. Therefore, if there is a small disturbance in the probability $P(t,\vec{N})$ of just one of the states $\vec{N}$ at some time *t*, then by a short time later *t* + *dt* this initial disturbance can only have affected a small number of states, the immediate neighbours of $\vec{N}$. After a second short interval of *dt*, the neighbours’ neighbours are also affected: and so on. Dis*t*urbances in the probabilities evolve by propagating outward from where they are introduced, which is very similar to how waves propagate. This motivates our approach: by choosing a representation where the distribution $P(t,\vec{N})$ is a smooth traveling wave Ψ instead of an infinitely large set of discrete values, we can analyse it using the same conceptual machinery that is used to describe wave propagation in applied mathematics and physics.

Briefly, our approach will be to restate (1) in terms of the differentiable function Ψ, and note that we can construct solutions by retracing “rays” from the initial conditions. This construction is possible because the system turns out to be hyberbolic: it has a certain causal structure, so that changes propagate in a “wavelike” way. By choosing an efficient algorithm for the “ray tracing” step, we can construct a valid distribution *P* and thus find $S_{f}$, which will let us compare this model to epidemiological data [5].

We will now briefly review Gillespie’s algorithm, before describing the formal solution of equation (1) via the method of characteristics, and then describe two numerical schemes based on it. The second scheme, described in 2.3.4, is novel and fast.

### 2.2 Gillespie’s stochastic simulation algorithm

Gillespie’s stochastic simulation algorithm provides a “brute force” method for estimating the distribution $P(t,N_{0},...)$ in (1), and is widely used to simulate biological processes and chemical reaction networks. The probability for each reaction to fire in a given time interval depends on the populations of different chemical species and the rate coefficients of different reaction pathways. The most important property of the Gillespie algorithm is that it provides an exact solution of the master equation (1) that describes the dynamics of the system, in the sense that the trajectories the algorithm produces are random samples of the probability distribution that solves the master equation (1) [31,32].

We define the state of the populations in the system to be $\vec{N}:=(N_{0},N_{1},...,N_{p})$ with each $N_{i}$ the population of species *i*; we index the reaction pathways with integers *j* in 0≤j≤k; and following D. Gillespie, let the rate of each reaction pathway *j* be $a_{j}(\vec{N})$ [31,32]. D. Gillespie’s stochastic simulation algorithm can then be described as follows:

1. Initialisation: set the time *t* = 0, set the populations $\vec{N}$ to their initial values $\vec{N}={\vec{N}}_{0}$, and initialise the random number generator.
2. With the system in state $\vec{N}$, evaluate every $a_{j}(\vec{N})$ and their sum $\Gamma =\sum_{j=0}^{k}a_{j}(\vec{N})$.
3. To decide which reaction *r* will happen, generate a uniform random number X∈[0,Γ]. The reaction *r* is the smallest integer that satisfies

$$\begin{array}{c} \sum_{j=0}^{r}a_{j}(\vec{N})<X \end{array}$$ (2)

This method of choosing *r* guarantees that the probability that reaction channel *r* is chosen this step is equal to $a_{r}(\vec{N})/\Gamma$.

4. Compute the time τ until the next reaction by generating a uniform random number Y∈(0,1], and computing

$$\begin{array}{c} \tau =\frac{1}{\Gamma }\ln\left(\frac{1}{Y}\right) \end{array}$$ (3)

5. Update the state $\vec{N}$ according to the reaction *r*, and increment *t* by τ.
6. Iteration: return to step 2 until the termination condition has been met. Example conditions are that the time *t* reaches some maximum value $t_{max}$, or a specific population $N_{i}$ reaches a maximum value.

In each iteration, only two calls to the random number generator are required.

While Gillespie’s algorithm is guaranteed to provide random samples of the master equation, carrying out enough replicates to get an accurate estimate of any of the probabilities will be very slow – essentially limited by the central limit theorem [30]. This motivated the search for faster methods. We will now outline one semi-analytical approach that can be used to solve this problem.

### 2.3 Analytical approach: The wave representation and formal solution

#### 2.3.1 Definitions.

First, we define the generating function Ψ to be the expected value of $\prod_{j\in V}q_{j}^{N_{j}}$ given a vector of conjugate coordinates $\vec{q}=(q_{0},q_{1},...)\in {\mathbb{C}}^{n}$ [33].


$$\begin{array}{c} \Psi (t,q_{0},q_{1},...,q_{j},...)=𝔼\left[\prod_{j\in V}q_{j}^{N_{j}}\right]=\sum_{\vec{N}}\prod_{j\in V}q_{j}^{N_{j}}P(t,N_{0},\cdots ,N_{j},\cdots ) \end{array}$$ (4)

where the sum runs over all possible values of the populations $\vec{N}:=(N_{0},N_{1},\cdots ,N_{j},\cdots )$. That is, $0\leq N_{j}<\infty$, for all j∈V. This is just the Fourier transform of the probability distribution $P(t,N_{0},...)$ with respect to the variables $N_{j}$ [33]. The variables $q_{j}$ are conjugate to the populations $N_{j}$, forming Fourier duals in a similar sense to frequency and time, or wavenumber and space (see also section 2.1, “Intuition”).

The probability $P(t,N_{f}=0,N_{f^{\prime }}=0)$ that the populations of two chosen final nodes *f* and $f^{\prime }$ are both zero at age *t* can then be expressed in terms of Ψ:


$$\begin{array}{c} \Psi (t,1,1,...,q_{j}=1,...,q_{f}=0,q_{f^{\prime }}=0)=\sum_{\vec{N}}P(t,N_{0},\cdots ,N_{j},\cdots ,N_{f}=0,N_{f^{\prime }}=0)=P(t,N_{f}=0,N_{f^{\prime }}=0) \end{array}$$ (5)

i.e., set $q_{f}=0$ for every node *f* that is unoccupied, and $q_{j}=1$ for every other node. More tersely, $q_{j}=1-\delta_{jf}-\delta_{jf^{\prime }}$, with $\delta_{ij}$ the Kronecker delta. In general, the probability that all nodes $f^{\prime }$ in a subset *X* are unoccupied at age *t* can be expressed as:


$$\begin{array}{c} P(t,N_{f^{\prime }}=0\;\forall f^{\prime }\in X)=\Psi (t,q_{0},...,q_{j},...) \end{array}$$ (6)

where $q_{j}=0$ if j∈X, and $q_{j}=1$ otherwise. This allows us to compute the diagnosis-free survival curve $S_{f}$:


$$\begin{array}{c} S_{f}(t)=\frac{\Psi (t,{\vec{q}}_{F})}{\Psi (t,{\vec{q}}_{F-f})} \end{array}$$ (7)

where the coordinate vectors ${\vec{q}}_{X}$ are shorthand for:


$$\begin{array}{c} {\vec{q}}_{F}:=q_{j}=0\;if\;j\in F,\;q_{j}=1\;otherwise \end{array}$$ (8)

and


$$\begin{array}{c} {\vec{q}}_{F-f}:=q_{j}=0\;if\;j\in F\;and\;j\neq f,\;q_{j}=1\;otherwise \end{array}$$ (9)

respectively. See Appendix A in S1 File for details of the definition of $S_{f}$.

When recast in terms of the generating function Ψ, equation (1) becomes the partial differential equation


$$\begin{array}{c} \frac{\partial \Psi }{\partial t}=ℋ\Psi \end{array}$$ (10)

where the hyperbolic differential operator ℋ (or “Hamiltonian”) is


$$\begin{array}{c} ℋ=\sum_{j\in V}X_{j}(\vec{q})\frac{\partial }{\partial q_{j}}+Y(\vec{q}) \end{array}$$ (11)

where


$$\begin{array}{cc} X_{j}(\vec{q}) & :=\alpha_{j}(q_{j}-1)q_{j}+\beta_{j}(q_{j}^{-1}-1)q_{j}+\sum_{k\in V}\mu_{jk}(q_{k}-1)q_{j}\\ Y(\vec{q}) & :=\sum_{j\in V}\kappa_{j}(q_{j}-1) \end{array}$$ (12)

The resemblance between equation (10) and others in mathematical physics is not a coincidence, and has been discussed by other authors [34].

The PDE form (10) is also infinite dimensional, like the original ODE hierarchy of the Kolmogorov forward equations. Since the ODE hierarchy is infinite dimensional, and the PDE is equivalent, they must both be infinite dimensional in some sense. To solve the system in either form, the system must be simplified or truncated. Different ways of truncating the system are discussed by S. Winkelmann and C. Schütte’s book and by R. Grima’s review [18,26]. Truncating the infinite hierarchy of ODEs is analogous to approximating the derivative operator in the PDE form with a finite difference approximation.

The system of equations (10) has a key property: it is hyperbolic. Hyperbolic systems of differential equations have the property that initial data propagate at a finite speed (which may vary locally) [35]. For the four reaction types we consider, system (10) is hyperbolic as long as the rate coefficients are positive and all the reactions are first- or zeroth-order. It does not matter that the reactions have specific stoichiometries: being a first-order reaction network is sufficient for hyperbolicity in our case. However, we don’t claim this is a *necessary* condition for hyperbolicity: at least some second-order reactions are also hyperbolic, but these systems are outside the scope of the generalised MSCE models considered here.

Hyperbolic partial differential equations are in a specific sense “wavelike”, in that initial data and disturbances propagate around at a finite speed: we can therefore construct solutions by looking backwards along the “rays” in the direction from which the waves proagate. This is the motivation for constructing solutions by the method of characteristics [35].

#### 2.3.2 Theorem 1: Construction of solutions.

A general solution to (10) is given by


$$\begin{array}{c} \Psi (t,q_{0},q_{1},...)=\left(\prod_{j\in V}\gamma_{j}(\sigma =t{)}^{Z_{j}}\right)e^{\int_{\sigma =0}^{t}Y(\vec{\gamma }(\sigma ))d\sigma } \end{array}$$ (13)

where the characteristics $\gamma_{j}$ are solutions of the initial value problem


$$\begin{array}{cc} \frac{d\gamma_{j}}{d\sigma } & =X_{j}(\vec{\gamma }),\\ \gamma_{j}(\sigma =0) & =q_{j} \end{array}$$ (14)

and the vector field $\vec{X}$ and scalar field *Y* are defined to be


$$\begin{array}{c} X_{j}(\vec{\gamma }):=\alpha_{j}(\gamma_{j}-1)\gamma_{j}+\beta_{j}(\gamma_{j}^{-1}-1)\gamma_{j}+\sum_{k\in V}\mu_{jk}(\gamma_{k}-1)\gamma_{j} \end{array}$$

and


$$\begin{array}{c} Y(\vec{q})=\sum_{j\in V}\kappa_{j}(q_{j}-1) \end{array}$$

and $\vec{\gamma }$ is shorthand for $\left(\gamma_{0},\gamma_{1},\cdots ,\gamma_{j},\cdots \right)$.

Values of Ψ can thus be computed for any given set of initial conditions $\vec{q}$. As an immediate corollary, this allows $S_{f}(t)$ to be calculated: the relevant initial conditions are given by (5), with $q_{j}=1$ for all j≠f, and $q_{f}=0$. Hence,


$$\begin{array}{c} P(t,N_{f}=0)=\Psi (t,q_{0}=1,q_{1}=1,...,q_{f}=0) \end{array}$$ (15)

For a proof, see appendix C.

#### 2.3.3 Numerical integration schemes based on Kolmogorov forward equations.

The formal solution (13) via the method of characteristics opens up many possibilities for numerical methods [29]. In principle, any numerical integration scheme that is appropriate for systems of ODEs can be used to solve (13), and thus give a numerical approximation for $S_{k}(t)$. As these methods can be expected to be “fast” in comparison to sampling with Gillespie’s algorithm, and are based on Kolmogorov forward equations instead of Kolmogorov backward equations (which are used to solve MSCE models by reduction to Gauss-Konrod quadrature – see [4,5,8]), we will refer to these as “fast forward” methods.

An early proposal due to D. Quinn relies on the method of characteristics to reduce the dimensionality of the system, and can also be used to find *S*(*t*) in MSCE models on path graphs faster than random sampling. However, as the desired error tolerance ϵ decreases, the run-time of Quinn’s method increases as $T\sim \epsilon^{-2}$: asymptotically, it is just as inefficient as random sampling, and was never widely adopted for this problem. On the other hand, Quinn’s method does not assume constant coefficients, so might be used as a starting point for the development of future methods. For a detailed analysis, see appendix D.

#### 2.3.4 Constant coefficients: A one-pass method.

Inspecting the formal solution (13) of the Kolmogorov forward equations immediately suggests other numerical schemes along the lines of Quinn’s. We will describe a simple implementation based on *improved* Euler integration: more sophisticated schemes are obviously possible. However, when the coefficients $\left\{\alpha_{k},\beta_{k},\kappa_{k},\mu_{jk}\right\}$ are independent of time, a more radical optimisation of Quinn’s method is possible.

Consider what happens to (13) when *t* is replaced by t+Δt, where Δt is a finite time step. Equation (13) is valid at all times, so it must be true that


$$\begin{array}{c} \Psi (t+\Delta t,q_{0},q_{1},...)=\left(\prod_{j\in V}\gamma_{j}(\sigma =t+\Delta t{)}^{Z_{j}}\right)e^{\sum_{j\in V}\int_{\sigma =0}^{t+\Delta t}\kappa_{j}(\gamma_{j}(\sigma )-1)d\sigma } \end{array}$$ (16)

Each characteristic solves (14), so


$$\begin{array}{c} \gamma_{j}(t+\Delta t)=\gamma_{j}(t)+\int_{t^{\prime }=t}^{t+\Delta t}X_{j}dt^{\prime } \end{array}$$ (17)

which can be computed with an appropriate numerical integration procedure, for example improved Euler integration:


$$\begin{array}{cc} \tilde{\gamma_{j}} & =\gamma_{j}(t)+X_{j}(\vec{\gamma })\Delta t\\ \gamma_{j}(t+\Delta t) & \approx \gamma_{j}(t)+\frac{\Delta t}{2}(X_{j}(\vec{\gamma })+X_{j}(\tilde{\vec{\gamma }})) \end{array}$$ (18)

(or a relative such as a higher-order Runge-Kutta, etc.).

The remaining term can also be approximated numerically. For brevity, call


$$\begin{array}{c} w(t):=\sum_{j\in V}\int_{\sigma =0}^{t}\kappa_{j}(\gamma_{j}(\sigma )-1)d\sigma \end{array}$$ (19)

and notice that


$$\begin{array}{cc} w(t+\Delta t) & =w(t)+\sum_{j\in V}\int_{\sigma =t}^{t+\Delta t}\kappa_{j}(\gamma_{j}(\sigma )-1)d\sigma \\ & \approx w(t)+\Delta w+𝒪(\Delta t^{3}) \end{array}$$ (20)

where the latter approximation integrates w(t+Δt) using improved Euler integration. The errors in this approximation must be of the same order as the errors in the integration of the characteristics $\vec{\gamma }$ in equation (18). Equation (16) can now be written


$$\begin{array}{c} \Psi (t+\Delta t,q_{0},q_{1},...)=\left(\prod_{j\in V}\gamma_{j}(\sigma =t+\Delta t{)}^{Z_{j}}\right)e^{w(t)+\sum_{j\in V}\kappa_{j}(\gamma_{j}(t)-1)\Delta t}+𝒪(\Delta t^{3}) \end{array}$$ (21)

Therefore, to calculate the value of Ψ at time t+Δt, we only need to know what the values of $\vec{\gamma }(t)$ and *w*(*t*) were. This shows that the generating function $\Psi (t,\vec{q})$ can be computed numerically, by applying a time-stepping procedure to the characteris*t*ics $\vec{\gamma }$ and the weight *w*(*t*). A concrete implementation in pseudocode might be:

1. Choose and fix a time step Δt and maximum time $t_{max}$.
2. Initialise *t* = 0, *w* = 0 and $\gamma_{j}=q_{j}$ for all *j*.
3. Update $\gamma_{j}$ for all *j* with an improved Euler step.
4. Update $w=w+\sum_{j\in V}\kappa_{j}(\gamma_{j}(t)-1)\Delta t$.
5. Update t=t+Δt.
6. Compute $\Psi (t,\vec{q})=\left(\prod_{j\in V}\gamma_{j}(t{)}^{Z_{j}}\right)e^{w}$.
7. If *t* is less than $t_{max}$, go to 3. Otherwise, exit.

The only dynamical quantities that need to be stored and updated are *t*, the weight *w*, and the characteristics $\gamma_{j}$. A new value of $\Psi (t,\vec{q})$ may be computed and returned at every time step. Only one set of characteristics need to be computed between σ=0 and σ=t to get a complete set of values for Ψ. This scheme terminates after $R=\lceil t_{max}/\Delta t\rceil \sim \Delta t^{-1}$ iterations. This is asymptotically much faster than Quinn’s method – requiring 𝒪(R) operations instead of $𝒪(R^{2})$.

More sophisticated schemes are easy to imagine. For example, improved Euler integration of the characteristics $\gamma_{j}$ could be replaced by an adaptive time step or one of a family of higher-order Runge-Kutta methods.

### 2.4 Application to statistical inference: Maximum likelihood optimisation

The fast and efficient computation of survival curves $S_{f}(t)$ suggests an application to statistical inference: namely, can this be used to estimate parameters of graphical MSCE models from real datasets? This was not feasible with random sampling [10,11,15,17]. In this section, we demonstrate such a regression algorithm using a simulated dataset [6], and study its performance.

**Fig 1:**
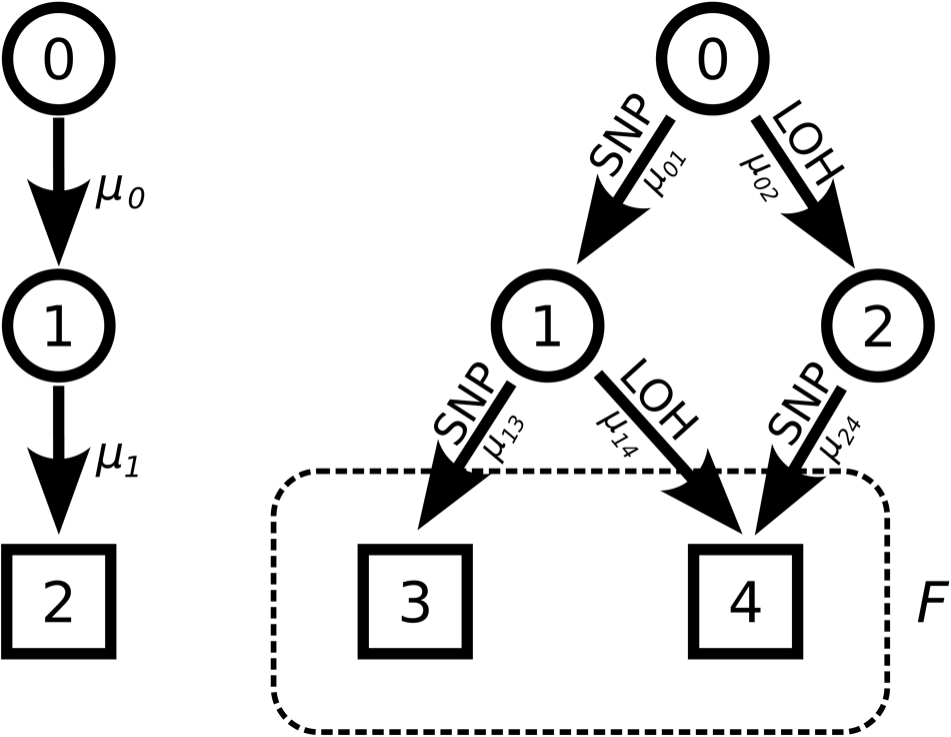
Left: an example of a two-step multi-stage clonal expansion model (MSCE) [3,6]. Right: a generalised MSCE on a directed acyclic graph with five nodes, with the end nodes (squares) in the set *F* labeled. This shows how two different mechanisms of tumour suppressor loss can be resolved in a graphical model. The two mechanisms in question are single-nucleotide variants (SNPs) on different alleles, and loss of heterozygosity (LOH) on the locus. The different end nodes in the set *F* correspond to different clonal copy number alterations in the resulting tumour: tumours of type 3 will have two mutated copies of the tumour suppressor, whereas those of type 4 will have one mutated copy and loss of heterozygosity on that locus.

To generate the dataset, we will assume the simple model of tumour suppressor loss from above (see Fig 1). To simulate the appearance of a tumour in a reference population of size *N*, *N* runs are performed with the Gillespie algorithm, with a known set of model parameters $\theta_{true}=\{\alpha_{j},\beta_{j},\kappa_{j},\mu_{jk}\}$, and the initial condition that every cell is initially on node 0. Cells on nodes 3 or 4 are neoplastic. The biological interpretation of the end node is whether or not the tumour presents with clonal LOH. In a more general model, the end node can represent what pattern of copy-number alterations (CNAs) is clonal in the resulting neoplasm [10,19].

If, in a given run, no cells arrive at either final node 3 or 4, this patient is considered not to have developed a primary tumour, and the age and type are not recorded. Thus, only a subset *m* of the *N* individuals in the simulated reference population will develop a tumour. The dataset therefore consists of a set of *m* < *N* observations, which consist of ages $t_{i}$ and end nodes/tumour types $f_{i}$, and the size *N* of the reference population. Once this dataset has been generated, the statistical problem is to find the best possible underlying parameters $\theta =\{\alpha_{j},\beta_{j},\kappa_{j},\mu_{jk}\}$. The most general way to achieve this is a maximum likelihood approach [13]. We will not attempt to infer the number of vertices on the underlying graph *G*, or the initial precursor cell population *Z*_0_.

**Fig 2:**
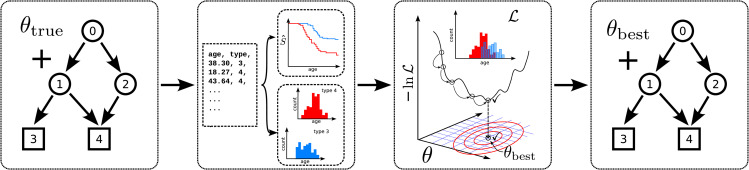
A figure describing the software framework that tests parameter inference by maximum likelihood estimation on a simulated longitudinal study. 1: A ground-truth model is proposed with known parameters $\theta_{true}$, and the Gillespie algorithm is used to generate a set of ages and types of diagnosed primary tumours, simulating a longitudinal clinical study. 2: the data set can be represented either with empirical estimators $\hat{S}(t)$ or a set of incidence histograms $I_{k}(t_{i})$. 3: the incidence in the simulated study is used to evaluate a likelihood function −lnℒ(θ) with our new fast forward method. This likelihood is then minimised using a combination of brute force and gradient descent, although other approaches are possible. 4: the final output of the harness is a set of estimated parameters $\theta_{best}$, which may then be compared to the ground truth parameters $\theta_{true}$. See section 3.

Once generated, the simulated dataset can be represented as a set of histograms, with one histogram for each tumour type *f* (see Fig 2). In each histogram, the data is binned according to age. The range of ages is split into uniform bins of width Δt: the $i^{th}$ bin has a start age $t_{i}$, and a count $k_{i}$. To compute the likelihood of this set of observations, a curve $S_{f}(t)$ is computed given a set of parameters θ. The probability (or discrete hazard) $h_{fi}$ that an individual will develop a primary tumour of type *f* in the $i^{th}$ bin, which covers the interval $\left[t_{i},t_{i}+\Delta t\right]$, is


$$\begin{array}{c} h_{fi}=\frac{S_{f}(t_{i})-S_{f}(t_{i}+\Delta t)}{S_{f}(t_{i})} \end{array}$$

If $n_{i}\leq N$ individuals in a reference population of *N* individuals have survived to the $i^{th}$ bin without being diagnosed with a tumour of type *f*, the number *k* that develop a tumour of type *f* in between the ages of *t* and t+Δt will be binomially distributed. Following S. Johansen [36], the likelihood ℒ of observing $k_{i}$ cases in the $i^{th}$ age bin given θ, which includes ages between $t_{i}$ and $t_{i}+\Delta t$, is


$$\begin{array}{c} ℒ(\theta ,bin\;i)=h_{fi}^{k_{i}}(1-h_{fi}{)}^{n_{i}-k_{i}} \end{array}$$

up to an irrelevant combinatorial prefactor. The combined likelihood for the simulated dataset is therefore


$$\begin{array}{c} ℒ(\theta )=\prod_{f\in F}\prod_{i=0}^{\lfloor t_{max}/\Delta t\rfloor }h_{fi}^{k_{i}}(1-h_{fi}{)}^{n_{i}-k_{i}} \end{array}$$ (22)

which corresponds to the likelihood for the Kaplan-Meier estimator $\hat{S}$ [36].

Our regression algorithm is then just a simple numerical method that maximises the above likelihood: we tested to see if it could infer the parameters $\theta_{true}$ used to generate a synthetic dataset. Briefly, after choosing a good start point with an initial round of brute force exploration, the parameters θ are varied using gradient descent, with the objective function defined to be −lnℒ from (22). Explicitly:


$$\begin{array}{c} -\ln ℒ(\theta )=-\sum_{f\in F}\sum_{i=0}^{\lfloor t_{max}/\Delta t\rfloor }\left[\ln(h_{fi})k_{i}+\ln(1-h_{fi})(n_{i}-k_{i})\right] \end{array}$$ (23)

The best possible estimate found using this method (the “best guess”) will be denoted $\theta_{best}$.

## 3 Results: Algorithm performance and application to statistical inference

The value of a fast numerical scheme for estimating $S_{f}(t)$ becomes apparent when comparing the run-time to random sampling, the only method known to be exact for birth-death processes on arbitrary graphs. To compare the methods fairly, we must determine how many replicates for Gillespie is equivalent to a chosen time step for the new, “fast forward” method: a fair comparison should ask how long both methods take to run when they are comparably accurate. In section 3.1, we show that our new method is asymptotically much faster than random sampling (with either Gillespie’s algorithm or τ-leaping).

The relevant survival curves $S_{f}(t)$ can now be estimated so rapidly that it becomes computationally feasible to run thousands of simulations for many different parameters, and thus compute likelihood functions for realistic datasets. In section 3.3, we describe a framework that generates a dataset from a single stochastic simulation with a known set of parameters; and then uses the fast forward method to compute a likelihood function on that simulated dataset. Applying simple optimisation algorithms to the resulting likelihood functions then results in maximum likelihood estimates of the parameters used to generate the underlying dataset (see Fig 2), in a similar way to known methods in the literature [6,37]. This is described in detail in section 3.3, in which we find that this framework can infer the hidden “ground truth” parameters even for small sample sizes of 10−100, suggesting that despite some caveats this approach holds much promise for real clinical data.

### 3.1 Scaling of errors and speed with step size Δt

In the following, we will define the error ϵ in either method to be the root mean squared error along a given time interval $t\in \left[0,t_{max}\right]$.

**Fig 3:**
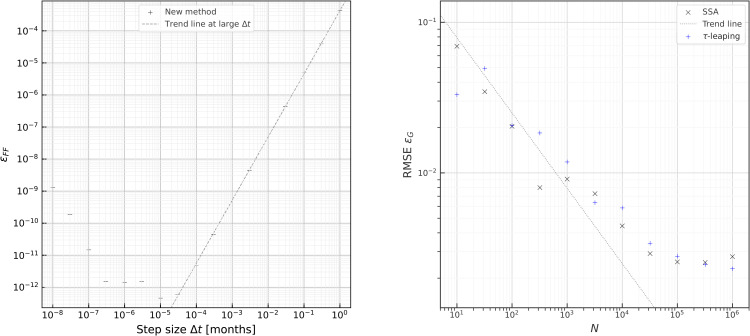
Plots showing error dependence of the Gillespie algorithm and our novel one-pass “fast forward” algorithm detailed in section 2.3.4. The model simulated was the 5-node model of tumour suppressor loss depicted in Fig 1. Left: The scaling of the global numerical errors in the “fast forward” method described in 2.3.4 as a function of step size Δt. The “floor” associated with round-off error is visible on the right, and on the left the upward $𝒪(\Delta t^{2})$ scaling of truncation error is apparent as Δt increases: the dashed line is the regression curve $\epsilon_{FF}\approx 1.27\times 10^{-3}\;\Delta t^{1.97}$. The global errors were estimated using Richardson extrapolation, comparing the computed value using a step size Δt to one at 0.5Δt [38]. Right: The scaling of global numerical errors for the Gillespie algorithm, defined as the mean squared difference between ${\hat{S}}_{k}$ for two simulations with different seed values. The dotted line is 0.5 *N*^-1/2^, estimated in section 3.1.

Random sampling estimates the underlying probability *P* of some event by performing *R* replicates of each simulation, and counting the number of replicates *k* in which the event occurred. The estimate of *P* is then just *P* = *k* / *R*. As the samples are independent and identically distributed, the count *k* will be binomially distributed. The associated standard deviation in the estimate *P* = *k* / *R* is therefore


$$\begin{array}{c} \sigma (P)=\sqrt{\frac{P(1-P)}{R}} \end{array}$$

and thus we expect the error to scale as $\epsilon_{G}=𝒪(R^{-1/2})$. This is indeed supported by simulations (Fig 3).

If a numerical integration scheme has a local error on the order of $𝒪(\Delta t^{p+1})$, then it will have a global error (over a finite interval) of $𝒪(\Delta t^{p})$ [38,39]. Since the local error in Heun’s method is $𝒪(\Delta t^{3})$, the global error $\epsilon_{FF}$ in the fast forward method’s estimate for $S_{f}(t)$ should be $𝒪(\Delta t^{2})$.

Numerically, the global error in a solver of ordinary differential equations can be estimated using Richardson extrapolation [38,40–42]. Fig 3 clearly shows that the error associated with the finite step size Δt scales as $𝒪(\Delta t^{2})$ (as expected).

For a fair comparison, we should choose Δt so that the targeted global error size ϵ for the fast forward method is the same order of magnitude as the global error for a set of *R* Gillespie algorithm simulations. That is,


$$\begin{array}{c} \epsilon_{G}=𝒪\left(\frac{1}{\sqrt{R}}\right)\approx \epsilon_{FF}=𝒪(\Delta t^{2}) \end{array}$$ (24)

The run time $T_{G}$ of the set of Gillespie algorithm simulations will be simply proportional to *R*, the number of runs performed, and so $T_{G}=𝒪(R)\sim 𝒪(\epsilon^{-2})$. On the other hand, the run time $T_{FF}$ of the fast forward method will scale as $T_{FF}=𝒪(1/\Delta t)\sim 𝒪(\epsilon^{-1/2})$. For a given error tolerance ϵ, we can expect


$$\begin{array}{c} T_{FF}\sim T_{G}^{\frac{1}{4}} \end{array}$$ (25)

This is what we mean by asymptotically faster: for any chosen error threshold ϵ, as ϵ decreases, the run-time $T_{FF}$ of the new method from 2.3.4 will increase much more slowly than $T_{G}$ will. For example, to reduce the error ϵ in *P*(*t*) by a factor of 1/100, $T_{FF}$ would only have to increase by a factor of 10, but $T_{G}$ would take 10^4^ times longer.

Possible generalisations of the fast forward method with higher-order numerical integrators can also be considered. When $\epsilon \sim \Delta t^{p}$, we have $T_{FF}\sim T_{G}^{\frac{1}{2p}}$, so that $T_{FF}$ should again scale with a small power of $T_{G}$.

### 3.2 Profiling and comparison of methods

For the Gillespie simulations, the survival curves $S_{k}$ were estimated using Aalen-Johansen estimators, as is appropriate for competing end states [36]. To estimate the error in the Gillespie approximation, two runs with the same sample size *N* were conducted using different seed values for the random number generator. The squared difference between the computed values for the resulting survival curves, ${\hat{S}}_{1}$ and ${\hat{S}}_{2}$, was rescaled by 1/2 and used as an estimate for the local mean squared error. The mean value of this local mean squared error was then used as a metric for the global error.

**Fig 4:**
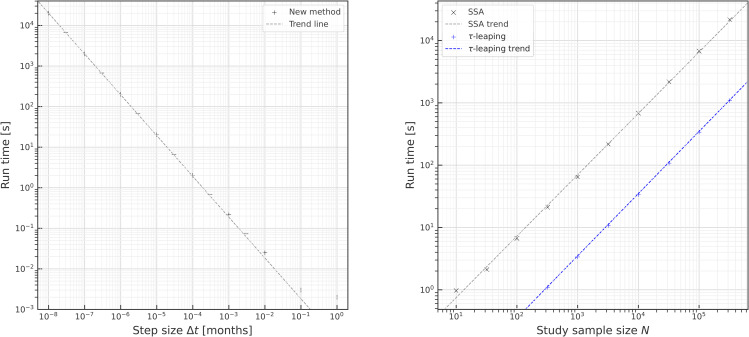
Plots of the real run time of the fast forward method as a function of Δt (left), and the run time of the stochastic method (right). The regression lines are: left, black: $T\approx 1.80\times 10^{-4}(\Delta t{)}^{-1.00}$, consistent with our argument that $T\sim \Delta t^{-1}$; right: trend lines for the Gillespie algorithm (black) and τ-leaping (blue). The Gillespie algorithm has $T\approx 7.77\times 10^{-2}N^{0.985}$, and τ-leaping has $T\approx 3.54\times 10^{-3}N^{0.998}$. This is also consistent with our argument that T∝N.

**Fig 5:**
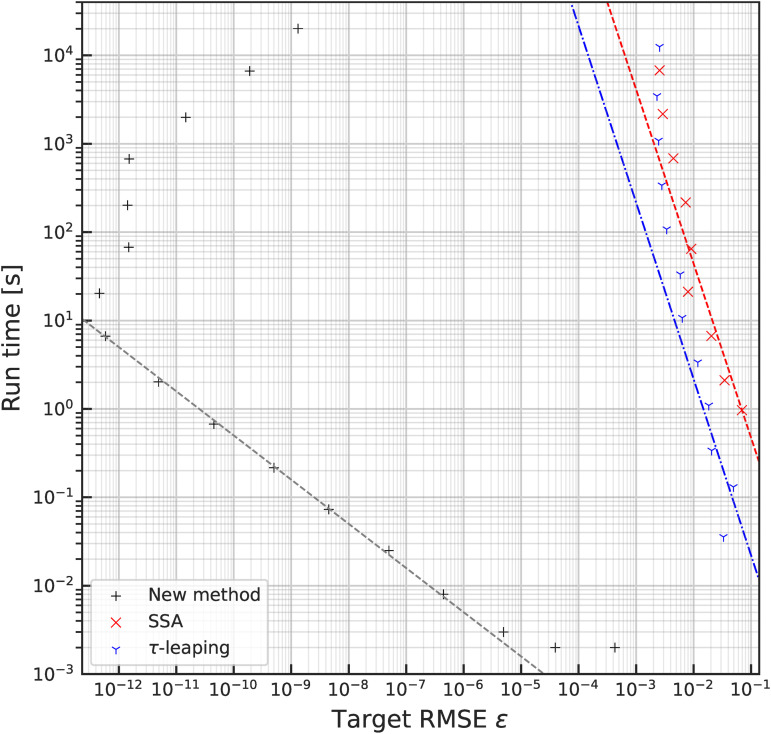
A plot showing how run time depends on desired root-mean squared error for both stochastic sampling with the Gillespie algorithm (red ×s), τ-leaping (blue *Y*s), and our new fast forward method (black +s). The $T\sim \epsilon^{-1/2}$ dependence of the fast forward method is apparent (black line), and a significant improvement on the $T\sim \epsilon^{-2}$ scaling of random sampling with either Gillespie’s algorithm (red line) or τ-leaping (blue line). Even for τ-leaping, the fast forward method is 10^7^ times faster for an error tolerance of $\epsilon =10^{-4}$, a dramatic improvement of seven orders of magnitude. Both the “fast forward” simulations and stochastic simulations were carried out on the University of Manchester’s high-performance computing cluster CSF3 *“Danzek”*.

Fig 3 shows that the global errors in the two methods being compared scale as predicted in section 3.1, and Fig 4 shows that the runtimes scale with the dependent variables in the expected way. Furthermore, Fig 5 shows that the scalings of runtime *T* with error tolerance ϵ of the two methods is also as predicted. It follows that the fast forward method will vastly outperform random sampling as the error threshold of interest ϵ is decreased, and this is indeed what is observed in Fig 5.

**Fig 6:**
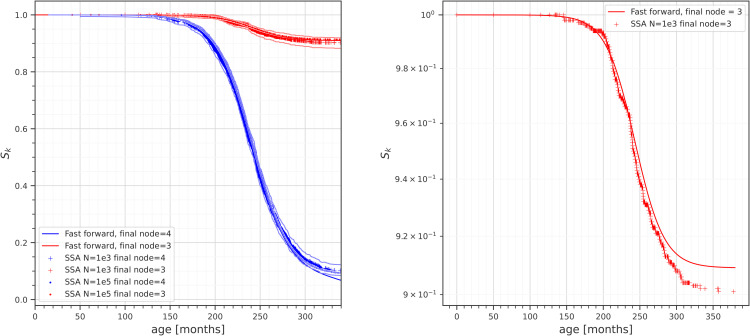
Left: a comparison of $\hat{S}$ curves for the tumour suppressor loss study generated by the Gillespie algorithm (+ signs: computed with *N* = 10^4^ replicates;· signs: computed with *N* = 10^6^ replicates), and the corresponding set of $S_{k}$ curves generated by our fast forward algorithm from section 2.3.4 (dashed); confidence intervals (shaded curves) are 95% confidence intervals around the empirical estimate $\hat{S}$ for *N* = 10^3^ replicates. Right, detail: an artifact causing deviations between the $\hat{S}$ and $S_{k}$ curves at late ages decreases as the sample size used for the stochastic simulations increases.

Comparing Aalen-Johansen estimators from Gillespie algorithm simulations to the $S_{k}$-curves from the fast forward method (Fig 6) shows excellent agreement across most of the range of the dataset, but reveals a surprising feature: the Aalen-Johansen $\hat{S}$ estimators and numerical *S*-curves visibly diverge at late ages. Close inspection reveals two reasons for this. The first is that this deviation occurs in a region where few data remain, in old age. It is in this neighbourhood where uncertainty in the values of the estimator is highest (see 95% confidence intervals in Fig 6). Secondly, as a non-parametric estimator, it cannot capture information about underlying trends that may continue after the last datum, as these are fundamentally parametric in nature: in this case, the exponential fall-off in old age. This exponential fall-off at late times is a well-studied and robust feature of MSCE models, so we are confident that the deviation here is an artifact of our non-parametric estimators’ intrinsic insensitivity to any parametric trend [5,6].

“Zooming in” on the problematic region confirms this: the numerical *S*-curve continues to decrease smoothly with age, whereas Aalen-Johansen estimators are biased towards flatness near the oldest datum. Increasing the number of replicates of the stochastic simulations improves agreement further, as visible in the detail on the right of Fig 6. Essentially, we believe the deviation of the empirical estimators is a result of increased uncertainty where data are sparse, and a bias towards flatness intrinsic to non-parametric estimators.

### 3.3 Application to statistical inference

The test framework described in Fig 2 was used to generate samples from a multi-stage model with known parameters, and then guess the underlying parameters for a range of different study sample sizes. This process mimics data collection from a longitudinal clinical study. The sample sizes considered varied from 10 to 10,000. We followed a general principle to test the framework on datasets that were realistically small, and targeted sample sizes of as small as 10. The statistical estimation scheme produced plausible order-of-magnitude estimates within two standard deviations of the ground-truth value without any prior information, but only for those sample sizes larger than *n* > 100 (see Figs 8–10). We considered this to be a success, showing that this inference scheme should be useful for many datasets available from longitudinal studies, even for relatively rare diseases.

**Fig 7:**
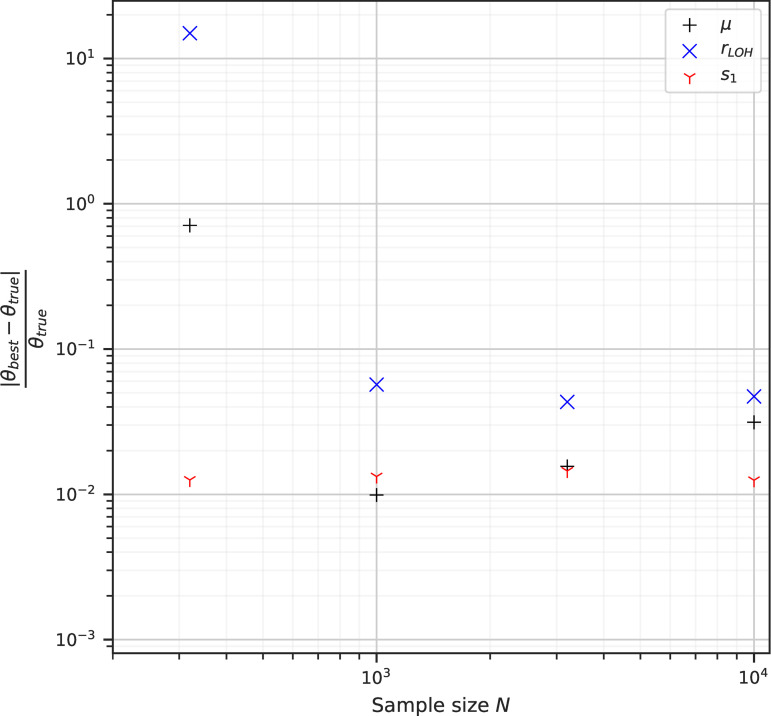
Plot showing relative errors in the test harness’ estimate of each parameter: μ, black +s; $r_{LOH}$, blue ×s; *s*_1_, red *Y*s. *s*_2_ is omitted as it proved to be unidentifiable: the other parameters do converge towards their ground truth values, but not monotonically. This prompted a deeper investigation of the structure of the likelihood function −lnℒ.

**Fig 8:**
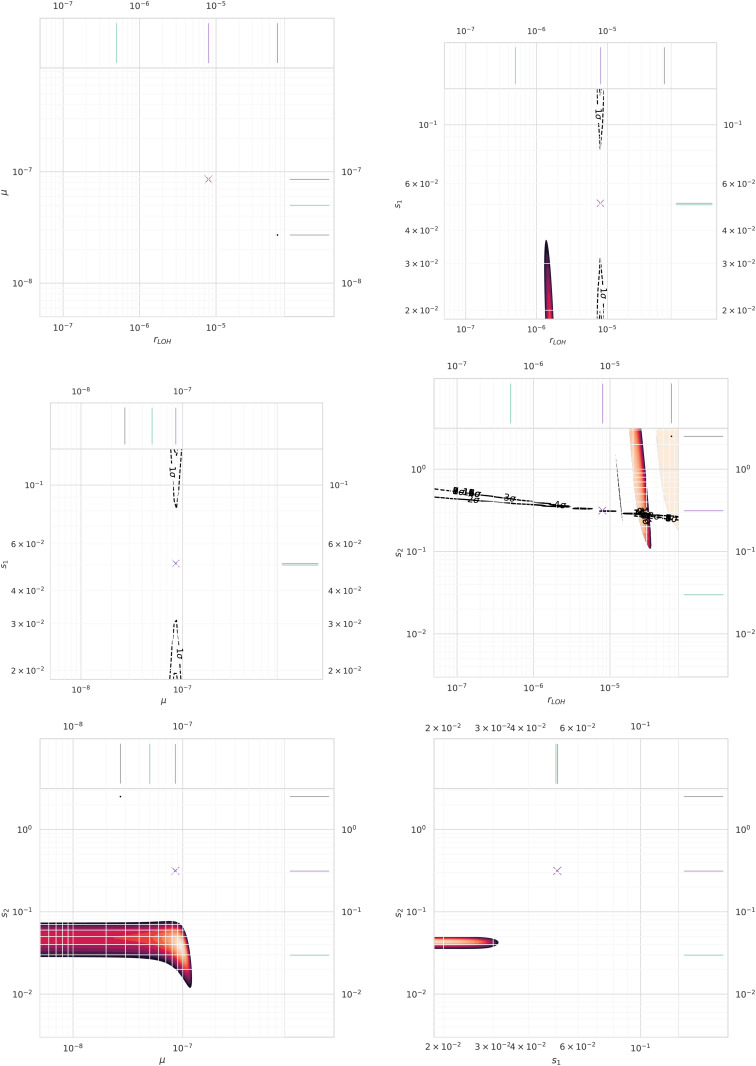
Results from the test harness, showing the parameter values for the ground truth (green +), best guess (purple x); a cross section of the likelihood function as a heat map (light orange to black), with likelihood levels that correspond to usual confidence intervals; confidence intervals derived from the Fisher information matrix (dashed ellipses), and a point cloud representing resampled best guesses (grey circles). Top left: μ and $r_{LOH}$; top right: $r_{LOH}$ and *s*_1_; centre left: μ and *s*_1_; centre right: $r_{LOH}$ and *s*_2_; bottom left: μ and *s*_2_; bottom right: *s*_1_ and *s*_2_. Sample size *N* = 320.

**Fig 9:**
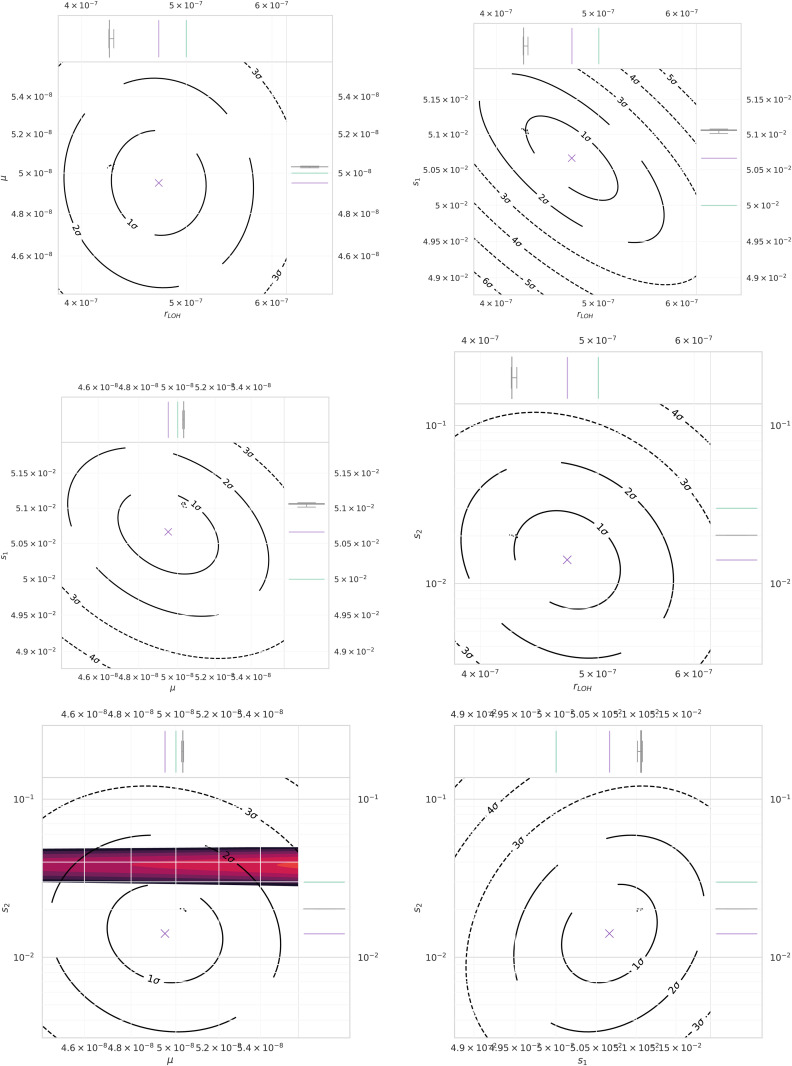
Results from the test harness, showing the parameter values for the ground truth (green +), best guess (purple x); a cross section of the likelihood function as a heat map (light orange to black), with likelihood levels that correspond to usual confidence intervals; confidence intervals derived from the Fisher information matrix (dashed ellipses), and a point cloud representing resampled best guesses (grey circles). Top left: μ and $r_{LOH}$; top right: $r_{LOH}$ and *s*_1_; centre left: μ and *s*_1_; centre right: $r_{LOH}$ and *s*_2_; bottom left: μ and *s*_2_; bottom right: *s*_1_ and *s*_2_. Sample size *N* = 1000.

**Fig 10:**
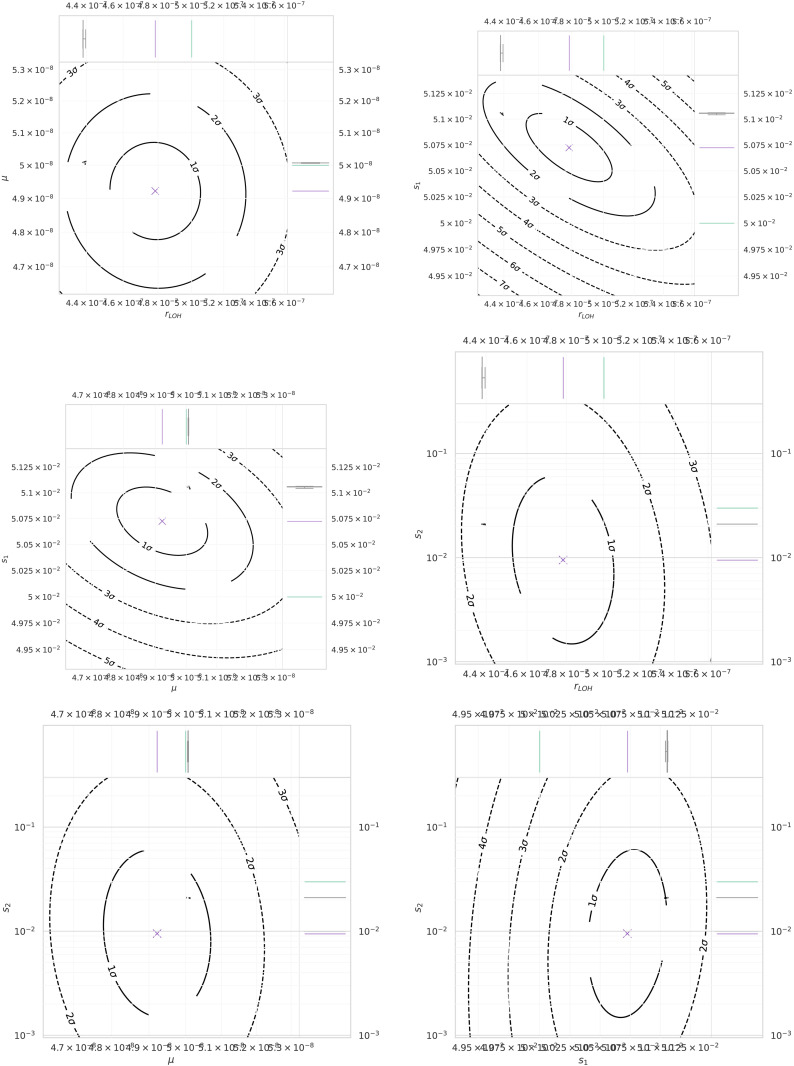
Results from the test harness, showing the parameter values for the ground truth (green +), best guess (purple x); a cross section of the likelihood function as a heat map (light orange to black), with likelihood levels that correspond to usual confidence intervals; confidence intervals derived from the Fisher information matrix (dashed ellipses), and a point cloud representing resampled best guesses (grey circles). Top left: μ and $r_{LOH}$; top right: $r_{LOH}$ and *s*_1_; centre left: μ and *s*_1_; centre right: $r_{LOH}$ and *s*_2_; bottom left: μ and *s*_2_; bottom right: *s*_1_ and *s*_2_. Sample size *N* = 3200.

To gauge the robustness of the estimates, the parameter estimates for each simulated study were also cross-validated using jack-knife resampling [43]. This method of cross-validation involves systematically removing points from datasets and iterating the inference algorithm: this demonstrated that the resulting maximum likelihood estimates were robust to missing datums and outliers (see Figs 8–10).

Our new “fast forward” method for evaluating $S_{k}$ is efficient enough that the likelihood function ℒ from 22 becomes relatively cheap to evaluate, which allows us to gain highly detailed information about its structure in hypothesis space. As well as the best guess from maximising the likelihood, it is possible to compute a Fisher information matrix (from the Hessian of −logℒ) near that best guess, which was used to generate analytical conference intervals using Wilks’ theorem [44]. However, it should be remembered that the maximum and Fisher information matrix only adequately characterise the distribution if the matrix is nonsingular and the distribution looks sufficiently like a normal distribution.

The main result of this section is that while maximum likelihood estimation does estimate values for the underlying parameters that are on the correct order of magnitude with minimal prior information, only the μ, $r_{LOH}$ and *s*_1_ parameters can be identified, with very large uncertainties in *s*_2_ (see Fig 7). This is consistent with prior work on identifiability in two-hit models [37]. We suspect that the underlying reason is that the likelihood function ℒ has an approximate symmetry that makes ℒ insensitive to *s*_2_, making this parameter unidentifiable [45,46].

To distinguish this from the known pitfalls of parameter inference in MSCE models, it must be noted that the initial precursor cell population *N*_0_ is not identifiable separately of μ when *N*_0_ is very large, a problem extensively studied in prior work [5,8,46]. Put briefly, this follows from a symmetry in the model dynamics and the stoichiometry of cell division: when death of wild-type cells is zero, and the population growth rate also zero, the initial population will be constant, and only the product $\mu N_{0}$ will be observable [5,8]. To address this, the population *N*_0_ is fixed to a known value rather than inferred, and only the mutation rates $\mu ,r_{LOH}$ and fitnesses $s_{1},s_{2}$ were varied during likelihood maximisation.

**Fig 11:**
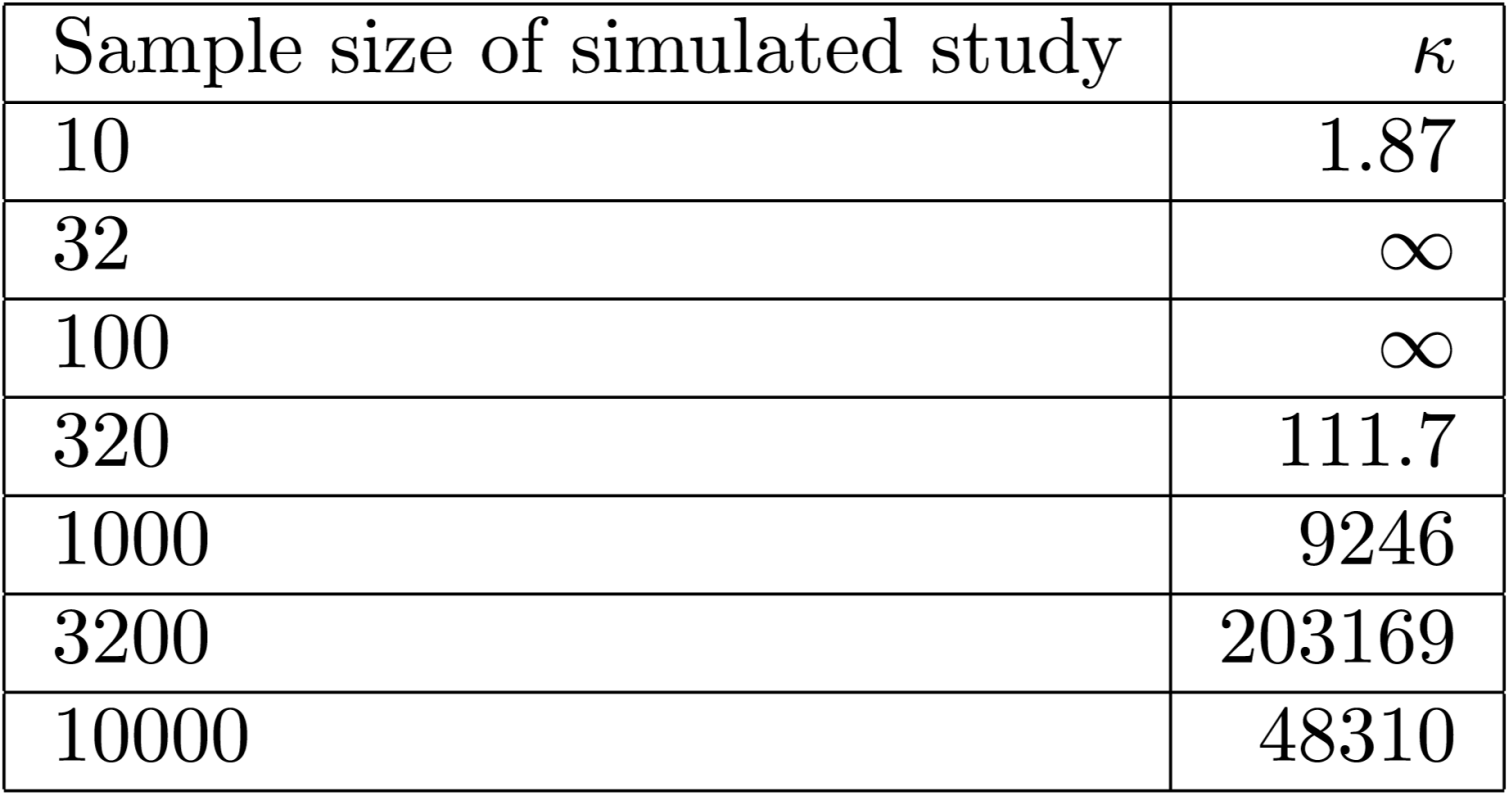
A table of condition numbers for each of the simulated clinical studies in the neighbourhood of the best guess. The condition number is defined as the ratio of the absolute values of the largest and smallest eigenvalues of the Fisher information matrix, $\kappa :={max}_{i}(|\lambda_{i}|)/{min}_{i}(|\lambda_{i}|)$. Condition numbers this high indicate issues with identifiability, we believe due to an approximate symmetry.

Even after this was taken into account, problems with likelihood maximisation remained. The condition numbers computed from the Fisher information matrix were large (see Fig 11), and in two cases the algorithm converged on a saddle point, indicating that the discovered best guesses lay in a region that was close to being singular. The Fisher information matrix was therefore ill-conditioned, which on the basis of prior work we suspect is due to a near-symmetry in the underlying model [37,45].

Instead of simply maximising the likelihood and studying local properties of ℒ, the function was sampled in detail in a neighbourhood around the best guess, and these samples rendered in 3D with custom video software (see supplemental information, S1 Video). This gave a highly detailed picture of the structure of ℒ. For each of the simulated clinical studies and estimates, six plots were made that showed the estimated and ground truth parameters with cross sections through ℒ. These plots revealed much fatter tails in ℒ than a normal distribution would have, as well as extreme broadness in the likelihood function in the *s*_2_ direction. This explains the problem with identifiability: the likelihood function is insensitive to *s*_2_, consistent with previous findings [37]. These plots are shown in Figs 8–10, including both confidence intervals derived from the Fisher information matrix and equivalent intervals in the likelihood function ℒ. These plots each show six cross-sections through the best guess $\theta_{best}$. Each cross-section shows how the likelihood varies with two of the four parameters.

Due to the issues with identifiability just mentioned, the plots for sample sizes of *N* = 32 and *N* = 100 were not informative as these tests failed to converge, and the plot with *N* = 10^4^ looked no more informative than the plots for smaller sample sizes. For this reason the tests of the inference harness with sample sizes *N* = 32,100 are not included in this manuscript, with all other tests shown as Figs 8–10. The plots for *N* = 10,10^4^ are presented as Figs L-M in Appendix G in S1 File.

There are apparent saddle points in the cross-sectional plots, which can indicate multiple local extrema. To study this, ℒ was sampled in a larger neighbourhood of the best guess, and volumetric ray casting was used to produce detailed 3-dimensional video renderings (included in the supplemental material, S1 Video). These renderings map $\mu ,r_{LOH},$ and *s*_1_ to the 3 spatial dimensions, mapping values of *s*_2_ to colour, and used normalised probabilities ℒ to indicate brightness [47]. The results revealed a complex, non-monotonic function that, in addition to the long narrow ridge that contained the best guess, had curious branching structures. As visible in the video renderings, the central, long, narrow ridge appears to double back on itself, with at least one additional maximum at high values of *s*_1_ and *s*_2_.

Despite the difficulties presented by these complex structures in the likelihood function, this method holds considerable promise for inferring mutation rates and other genomic parameters in relatively rare diseases. However, identifiability remains a problem: for *n* = 32 and *n* = 100, the minimisation algorithm converges on a saddle point, which we believe exists because of an approximate symmetry. The long, narrow ridge and other structures in the likelihood function are caused by the parameter *s*_2_ in particular being identifiable, which future research must try to address or mitigate.

## 4 Discussion

The fast algorithm presented in section 2.3.4 allows survival curves and first-passage times for clonal selection models to be evaluated rapidly and deterministically: it is always at least seven orders of magnitude faster than any practical method based on random sampling. This in turn enables mutation rates and fitness advantages during carcinogenesis to be estimated from simulated data using maximum likelihood methods, without the use of approximate Bayesian computation or related techniques. This computation of likelihood functions was not previously feasible. However, although the likelihood function for this model can be computed, much additional work remains to improve the identifiability of the model. We have shown using simulated clinical studies that the resulting risk models should be able to combine epidemiological and genomic data, correlating age with copy number alterations, or other genomic rearrangements. In the future, these models may be able to capture individualised risk factors: perhaps showing how rare variants or genomic signatures interact with patient age.

This class of model should permit better estimates of parameters of biological mechanisms, as well as improved forecasts of cancer incidence with age and risk factors, with clear applications in public health modelling. [10,11,17]. While the method described here was motivated by models of cancer incidence, the numerical scheme should also be applicable to any chemical reaction network that is first-order and has constant rate coefficients [29]. Although the “fast forward” method described in section 2.3.4 has key limitations – namely, for all reactions to be first order, and for the coefficients to be constant – it is clearly applicable to reactions with more than one product, which gives it a broader scope than known exact solutions [20,22–24].

It is also interesting to note that the various “classical” methods based on Kolmogorov backward equations typically have several generating functions $\Phi_{k}$ arranged in a hierarchy [5,15,16]. In contrast, both our “fast forward” method from section 2.3.4 and Quinn’s earlier fast forward method rely on only one generating function Ψ with multiple arguments. The analogous variables to $\Phi_{k}$ in our approach are the characteristic curves, $\gamma_{k}$ [4].

Our new method is limited to models with constant coefficients. Continued work must discover an efficient numerical integration scheme for evolution on graphs that is suitable for time-varying parameters $\theta (t)=\{\alpha_{j}(t),\beta_{j}(t),\mu_{jk}(t),\kappa_{j}(t)\}$. This will be necessary to study carcinogenic processes associated with mutagen exposure, chronic inflammatory conditions, or other environmental factors. Such factors are relevant to many important public health challenges, such as Barrett’s oesophagus, obesity, or smoking; so a fast method to handle non-constant parameters θ would be immediately useful and a natural next step for future research [6,13]. Quinn’s algorithm of 1989 (“Q89”) is one candidate, but our analysis in section 3.1 and contemporary reviews suggest that its runtime scales as $T\sim 𝒪(\epsilon^{-2})$, so it is not asymptotically efficient [8,12,14]. On the other hand, our work suggests that there may be a family of unexplored relatives to this algorithm with considerable optimisations, both regarding the rate of convergence of the numerical integrator and the amount of necessary caching. A hypothetical two-pass method like Q89 that replaced Euler integration with a fourth-order Runge-Kutta method should have a theoretical runtime complexity of $T\sim 𝒪(\epsilon^{-\frac{1}{2}})$. This would be a much more efficient method in the “fast forward” family that could be useful for a wider range of epidemiological studies than we have considered.

Nonetheless, we have established that survival probabilities $S_{f}(t)$ and hazard functions $h_{f}(t)$ for MSCE models on graphs can be computed arbitrarily accurately and very rapidly, asymptotically much faster than random sampling. The relevant probabilities can be computed so fast, in fact, that it becomes computationally feasible to perform maximum likelihood estimation, finding the model of best fit for a given clinical study. We look forward to deploying the statistical inference scheme on other projects with real data: in such cases, the assumption of constant parameters should be understood as resulting in effective parameters which are essentially averaged over the timescale of the study. More detailed inferences about dynamics must await more sophisticated models.

Our work also suggests the existence of a larger family of “fast forward” methods for studying stochastic processes on graphs, which may be efficient alternatives to existing methods. Similarly, the algorithm used for maximum likelihood estimation in section 3.3 could be replaced with many other optimisation algorithms. The choice of which numerical integration scheme to pair with which algorithm to best infer the parameters of cancer evolution from genomic epidemiology is a new, open question. In addition, now that likelihood functions for graphical MSCE models on data can be visualised in detail, additional research could study these functions’ symmetries and what new issues with identifiability may arise [37].

## Supporting information

S1 VideoS1_Video.webm.Supplemental videos: renderings of the likelihood function of the model in four dimensions (treated as 3 dimensions + colour). *x*-axis (red): μ, *y*-axis (green): $r_{LOH}$, *z*-axis (blue): fitness *s*_1_, colour: average *s*_2_ value. Each video corresponds to a different study sample size *N*. These videos were rendered with volumetric ray casting [47]. These video files can be played with VLC or a modern web browser. Sample size for S1_Video: 10 cases.(WEBM)

S2 VideoS2_Video.webm.μ$r_{LOH}$ Sample size for S2_Video: 32 cases.(WEBM)

S3 Video S3_Video.webm. Sample size for S3_Video: 100 cases.
(WEBM)

S4 Video S4_Video.webm. Sample size for S4_Video: 320 cases.
(WEBM)

S5 Video S5_Video.webm. Sample size for S5_Video: 1000 cases.
(WEBM)

S6 Video S6_Video.webm. Sample size for S6_Video: 3200 cases.
(WEBM)

S7 Video S7_Video.webm. Sample size for S7_Video: 10000 cases.
(WEBM)

S1 Fig The file S1_Fig.png is itself a graphical legend showing the numerical ranges on the axes of the video renders. (PNG)

S1 File S1_File.pdf: other supplemental information: Appendices A-F include proofs of various lemmas in the main manuscript, and Appendix G contains supplemental figures, being the results of the test harness for other sample sizes. (PDF)
