# Supplementary material for: A fast numerical integration scheme for clonal expansion processes on graphs
Source: PLoS Comput Biol. 2026 Aug 4;22(8):e1012784. doi: 10.1371/journal.pcbi.1012784 (PMC13436781; doi:10.1371/journal.pcbi.1012784)
Supplement: Supplementary file 1 [file pcbi.1012784.s009.pdf]

Supplemental information: appendices

A Appendix: definition of survival curve

There are four types of process in a generalised MSCE model: mutation, birth, and death obey first-order kinetics, and immigration obeys zeroth-order kinetics. These models include four types of process:

- Mutation (by asymmetric division): $j \to j + k$, at rate $\mu_{jk} N_j$
- Birth (by symmetric division): $j \to j + j$, at rate $\alpha_j N_j$
- Death: $j \to \emptyset$, at rate $\beta_j N_j$
- Immigration (to approximate mutations appearing in a large unobservable population [5]): $\emptyset \to j$, at rate κ_j

labelling different cell types j and k, and the numbers of these types N_j and N_k, respectively.

The key quantity the probability $S_f(t)$ that no primary tumour of type f has appeared by a given age t. From $S_f(t)$, the age-specific incidence of each subtype f can be derived. From $S_f(t)$, one can define the hazard function $h_f(t)$,

$$h_f(t) = -\frac{d \ln S_f(t)}{dt} \tag{26}$$

which models the age-related incidence [5].

In the case that the set of final nodes F has only one member, the relationship of the probability distribution $P(t, \vec{N})$ to the survival curve $S_f(t)$ is straightforward, and

$$S_f(t) = P(t, N_f = 0) \tag{27}$$

But in general, the set of final nodes F on the graph G may have more than one member. Unlike the case of path graphs, in which we can identify the probability to have no cancerous cells $P(t, N_f = 0)$ with the survival curve $S_f(t)$, when there are multiple end states the proper definition of S_f is more subtle. Each of the multiple end states in F represent different sets of clonal alterations in the initiated primary tumour: these mutations will be present in every descendant cell in the resulting tumour.

Models defined on arbitrary graphs admit cases in which more than one final subpopulation (say N_f and $N_{f'}$) are nonzero simultaneously. Biologically, this corresponds to a scenario in which a tumour of type f' arises after a primary tumour of type f is already present (or vice versa). This is not unrealistic, as multifocality is observed in some types of brain tumour [48]. However, it complicates the definition of $S_f(t)$, as the simple probability $P(t, N_f = 0)$ will include cases that are not primary tumours.

Recall that $S_f(t)$ should be the probability that a patient survives to age t without a diagnosis of a primary tumour of type f. Without loss of generality, we can assume that the probability that tumours of two distinct types $f \neq f'$ cannot arise simultaneously. The relevant probability, so that we count only primary tumours of type f, is the probability that the subpopulation $N_f = 0$, conditioned on all other final subpopulations being zero:

$$S_f(t) = P(t, N_f = 0 | N_{f'} = 0 \; \forall f' \in F : f' \neq f) \tag{28}$$

This counts only cases of type f where there is no existing tumour of type $f' \neq f$. These cases are precisely the primary tumours of type f.

By the definition of conditional probability $P(A|B) := P(A \cap B)/P(B)$, equation (28) implies

$$S_f(t) = \frac{P(t, N_f = 0 \cap \; N_{f'} = 0 \; \forall f' \in F : f' \neq f)}{P(t, N_{f'} = 0 \; \forall f' \in F : f' \neq f)}$$

Which is equivalent to

$$S_f(t) = \frac{P(t, N_{f'} = 0 \; \forall f' \in F)}{P(t, N_{f'} = 0 \; \forall f' \in F : f' \neq f)}$$

That is, compute $P(t, N_{f'} = 0, \dots)$ for all final nodes in F; then compute $P(t, N_{f'} = 0, \dots)$ for all final nodes f' *except for* f; and S_f is then given by the ratio.

B Appendix: note on more general stoichiometries

Other stoichiometries for mutation/migration reactions can also be considered. In general, a first-order reaction of the form

$$j \to mj + nk \tag{29}$$

will contribute a term proportional to

$$(q_j^{m-1} q_k^n - 1) q_j \frac{\partial}{\partial q_j} \tag{30}$$

to the Hamiltonian in equation (10). More general mass-action kinetics can also be considered in this framework: if we write $N_j := q_j \frac{\partial}{\partial q_j}$ for each reagent j and $\vec{\Delta} := (\Delta N_0, \Delta N_1, \dots) = \vec{N}(after\ reaction) - \vec{N}(before\ reaction)$ for the overall stoichiometric vector of the reaction, then the most general term for a reaction will be

$$\left(\prod_{j \in V} q_j^{\Delta_j} - 1 \right) \prod_{j \in R} \left(q_j \frac{\partial}{\partial q_j} \right) \tag{31}$$

where $R \subset V$ is the set of reagents for the reaction. Note that the various N_j operators commute [29, 34].

C Appendix: proof of theorem 1

The wave equation (10) can be placed into a conservation form along a vector field $\vec{X} = (X_0, X_1, \cdots, X_j, \cdots)$:

$$\frac{\partial \Psi}{\partial t} = \vec{X}(\vec{q}) \cdot \nabla \Psi + Y(\vec{q}) \Psi \tag{32}$$

This can be solved using the method of characteristics [49]. Briefly, we seek a parametrised family of curves $\tau(\sigma)$, $\vec{\gamma}(\sigma)$ for which

$$\frac{d\Psi}{d\sigma} = \frac{d\tau}{d\sigma} \frac{\partial \Psi}{\partial t} + \sum_{j \in V} \frac{d\gamma_j}{d\sigma} \frac{\partial \Psi}{\partial q_j} \tag{33}$$

and $\tau(0) = t, \vec{\gamma}(0) = \vec{q}$. An appropriate family of curves is

$$\frac{d\tau}{d\sigma} = -1$$
$$\frac{d\vec{\gamma}}{d\sigma} = \vec{X}$$

Clearly $\tau(\sigma) = t - \sigma$. The characteristics $\vec{\gamma} = (\gamma_0, \gamma_1, \cdots)$ will have a more complicated form.

Along these curves, equation (32) takes the form

$$\frac{d\Psi}{d\sigma} = -Y(\vec{\gamma}) \Psi \tag{34}$$

where

$$Y(\vec{\gamma}) = \sum_{j \in V} \kappa_j (\gamma_j - 1) \tag{35}$$

Integrating (34) and rearranging for $\Psi(\tau(0) = t, \vec{\gamma}(0) = \vec{q})$ yields

$$\Psi(\tau(0) = t, \vec{\gamma}(0) = \vec{q}) = \Psi(\tau(\sigma = t) = 0, \vec{\gamma}(\sigma = t)) \exp \left(\int_{\sigma=0}^{t} Y(\vec{\gamma}) d\sigma \right) \tag{36}$$

Given that the initial conditions are known,

$$\Psi(0, \vec{\gamma}(\sigma = t)) = \prod_{j \in V} \gamma_j(\sigma = t)^{Z_j} \tag{37}$$

substituting this results in the formal solution (13).

D Appendix: Non-constant parameters: Quinn's method

When the coefficients $\{\alpha_k(t), \beta_k(t), \kappa_k(t), \mu_{jk}(t)\}$ have an explicit time dependence, the following scheme by Quinn can be used [14]. Explicit time dependence is relevant to e.g. tobacco smoking and possibly inflammatory conditions linked to pre-cancer such as Barrett's oesophagus. In the following, the coefficients are assumed to have a specified functional form, and e.g. $\alpha_j(t)$ represents a function call.

For each value of t, new characteristic curves $\gamma_j(\sigma)$ (with initial conditions q_j) and weights $w = \int Y(\vec{\gamma}(\sigma))d\sigma$ are computed in a "two pass" procedure, using Euler integration to update the γ_j, and the rectangle rule to update the weights. These characteristic curves can then be substituted into (13), and corresponding values for $P(N_j = 0)$ computed.

1. Choose and fix a time step Δt.
2. For each time of interest t...
3. Initialise $\sigma = 0$, $w = 0$ and $\gamma_j = q_j$ for all j.
4. While $\sigma < t$:
5. Update $\gamma_j = \gamma_j + X_j \Delta t$ for all j. (Euler integration)
6. Update $w = w + Y(\vec{\gamma}(t))\Delta t$.
7. Update $\sigma = \sigma + \Delta t$.
8. Store the values $(\vec{\gamma}, \sigma)$.
9. Using the cached values of $(\vec{\gamma}, \sigma)$ compute $\int_{\sigma=0}^{t} Y(\vec{\gamma}(\sigma))d\sigma$ and hence $\Psi(t, \vec{q})$.

For any explicit time-stepping procedure with step size Δt, the number of operations to compute one of these characteristic curves will be $N = \lceil t_{max}/\Delta t \rceil$. To compute values of $P(N_j = 0)$ at all N ages in between $t = 0$ and $t = t_{max}$ will therefore require $\mathcal{O}(\Delta t^{-2})$ operations. This is explicitly described in Crump's review and Quinn's original paper [12, 14].

The lack of an asymptotic speed-up for this method is probably in part why interest in approaches based on characteristics waned during the 1990s, as Luebeck and Moolgavkar's work successfully reduced the problem to Gaussian quadrature [12].

E Appendix: asymptotic scaling and other parameters

Suppose the model's graph $G = (V, E)$ has some number of nodes $|V|$ [9]. The event rate of any stochastic simulation method will increase as the number of vertices on the graph increases, and thus so too will the number of operations and overall runtime. The same is true for the fast forward method, which visits each node once each time step. Thus, both methods scale like $T \sim |V|$, and the size of the underlying graph should not be relevant to the comparison.

The scaling of runtime with initial population size Z_0 is more interesting. As a larger population size results in a higher event rate, and the dynamics are linear (or first-order), the overall runtime of random sampling should scale like $T \sim Z_0$. An initial population that is twice as large will take twice as long to simulate. However, in the fast forward method (or any related method, like Quinn's 1989 method or MVZK quadrature) Z_0 appears only as an argument to a function call. Essentially, Z_0 is only used in a multiplication or exponentiation operation. This has no effect on the overall number of operations in any step. As a result, the runtime of fast forward methods (and related methods) is basically independent of population size, so $T \sim const.$ wrt. Z_0.

F Appendix: projection of level sets of the Fisher information matrix

Our statistical inference framework finds a global minimum of the negative log-likelihood $-\ln \mathcal{L}$. Futhermore, being able to efficiently evaluate the likelihood function means that we can also estimate the Fisher information matrix from the Hessian H of $\mathcal{L}$ near the best guess".

By Wilks' theorem, we can then generate confidence intervals by studying the level sets of H. Our parameter space $\vec{\theta} = (r_{LOH}, \mu, s_1, s_2)$ is four-dimensional. To visualise structures in this space, it is natural to ask what two-dimensional projections of these structures look like.

Consider the ellipsoid hypersurface E defined by

$$\Delta\vec{\theta}^T \cdot H \cdot \Delta\vec{\theta} = n^2 = \text{const.} \tag{38}$$

where n is an integer that indexes the confidence interval, and

$$\Delta\vec{\theta} = \vec{\theta} - \vec{\theta}_{best\ guess} \tag{39}$$

is a vector representing differences from the best guess, or minimum of $-\mathcal{L}$.

Without loss of generality, choose the first two components of the vector $\Delta\vec{\theta}$ as the $x-y$ plane onto which we will project an image of the level set defined by (38), and call these $\vec{x} = (x, y)$. Label the other components of $\Delta\vec{\theta}$ with $\vec{z} = (z_1, z_2)$. Thus, $\Delta\vec{\theta} = (x, y, z_1, z_2) = (\vec{x}, \vec{z})$. The $\vec{x}-$vector represents coordinates in the image plane S, and the $\vec{z}-$vector represents all other coordinates.

Everywhere on E, the surface normal to E must lie parallel to the image plane. So for both the z_1 and z_2 directions, we must have the following:

$$\nabla\left(\Delta\vec{\theta}^T \cdot H \cdot \Delta\vec{\theta}\right) \cdot \begin{bmatrix}0\\0\\1\\0\end{bmatrix} = 0 \tag{40}$$

$$\nabla\left(\Delta\vec{\theta}^T \cdot H \cdot \Delta\vec{\theta}\right) \cdot \begin{bmatrix}0\\0\\0\\1\end{bmatrix} = 0 \tag{41}$$

Now,

$$\nabla\left(\Delta\vec{\theta}^T \cdot H \cdot \Delta\vec{\theta}\right) = (H + H^T) \cdot \Delta\vec{\theta} = 2H \cdot \Delta\vec{\theta} \tag{42}$$

because H is symmetric.

Decompose H in the following way:

$$H = \begin{bmatrix} A & C \\ C^T & B \end{bmatrix} \tag{43}$$

and the two conditions (40) and (41) become

$$C \cdot \vec{x} + B \cdot \vec{z} = \begin{bmatrix}0\\0\end{bmatrix} \tag{44}$$

so everywhere on the boundary E, $\vec{z}$ and $\vec{x}$ must be linearly related (i.e. coplanar):

$$\vec{z} = -B^{-1} \cdot C \cdot \vec{x} \tag{45}$$

and thus

$$\Delta\vec{\theta} = \begin{bmatrix} \mathbb{I} \\ -B^{-1}C \end{bmatrix} \cdot \vec{x} \tag{46}$$

with $\vec{x} = (x, y)$; so

$$\Delta\vec{\theta}^T \cdot H \cdot \Delta\vec{\theta} = \vec{x}^T \cdot \left(\begin{bmatrix} \mathbb{I} \\ -B^{-1}C \end{bmatrix}^T \cdot H \cdot \begin{bmatrix} \mathbb{I} \\ -B^{-1}C \end{bmatrix} \right) \cdot \vec{x} \tag{47}$$

and finally

$$\begin{bmatrix} \mathbb{I} \\ -B^{-1}C \end{bmatrix}^T \cdot H \cdot \begin{bmatrix} \mathbb{I} \\ -B^{-1}C \end{bmatrix} = \begin{bmatrix} \mathbb{I} \\ -B^{-1}C \end{bmatrix}^T \cdot \begin{bmatrix} A & C \\ C^T & B \end{bmatrix} \cdot \begin{bmatrix} \mathbb{I} \\ -B^{-1}C \end{bmatrix} = A - C \cdot B^{-1} \cdot C^T \tag{48}$$

so to visualise the confidence region, the projection of the level set E given by (38) can be expressed purely in terms of the image plane coordinates $\vec{x} = (x, y)$ in the following way:

$$\vec{x}^T \cdot (A - C \cdot B^{-1} \cdot C^T) \cdot \vec{x} = \vec{x}^T \cdot (H/B) \cdot \vec{x} = n^2 = \text{const.} \tag{49}$$

that is, using the Schur complement H/B [50].

G Supplemental figures

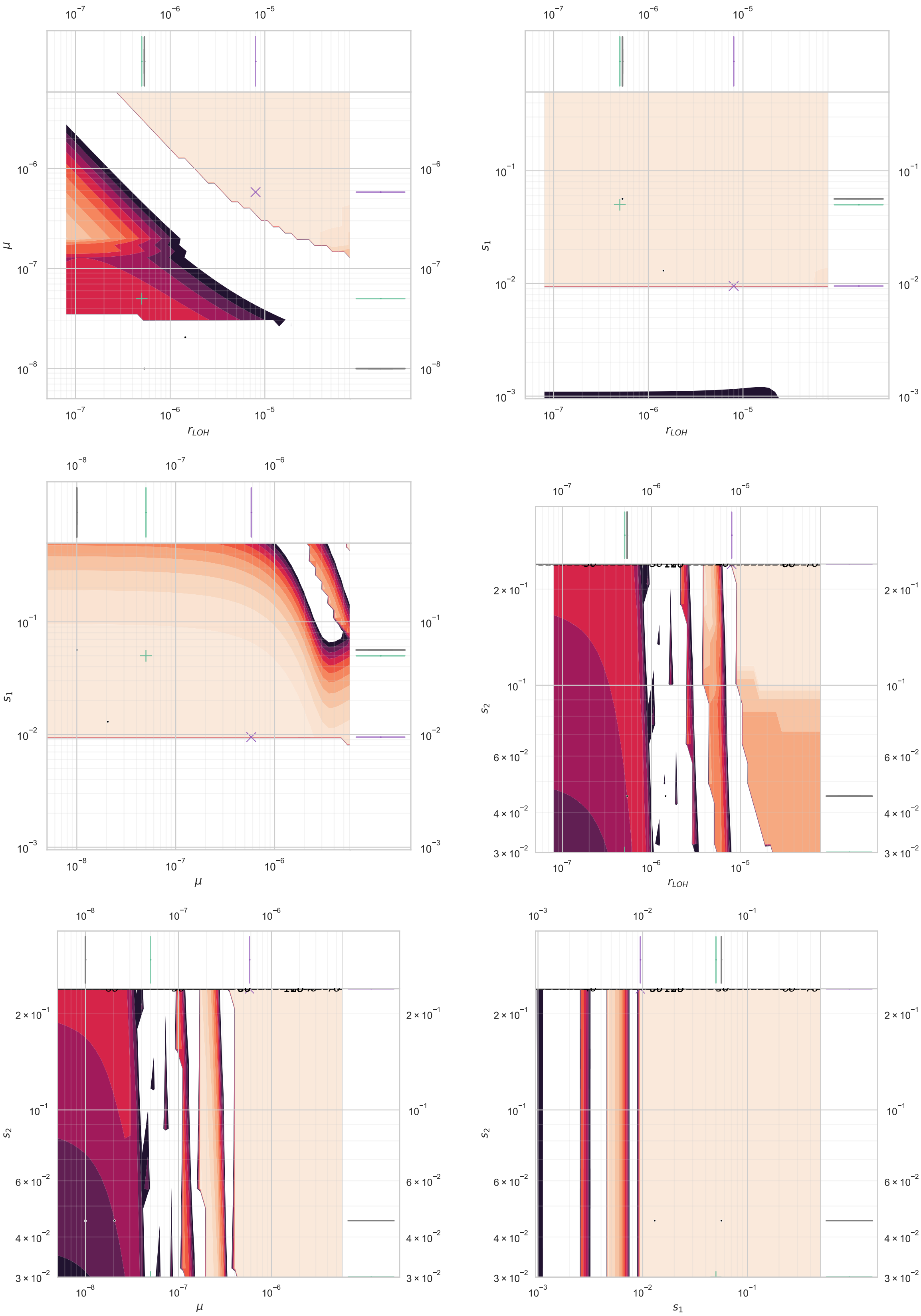

Fig L: Results from the test harness, showing the parameter values for the ground truth (green +), best guess (purple x); a cross section of the likelihood function as a heat map (light orange to black), with likelihood levels that correspond to usual confidence intervals; confidence intervals derived from the Fisher information matrix (dashed ellipses), and a point cloud representing resampled best guesses (grey circles). Top left: μ and r_{LOH}; top right: r_{LOH} and s_1; centre left: μ and s_1; centre right: r_{LOH} and s_2; bottom left: μ and s_2; bottom right: s_1 and s_2. Sample size $N = 10$. Note the numerical artifacts at large values of s_2.

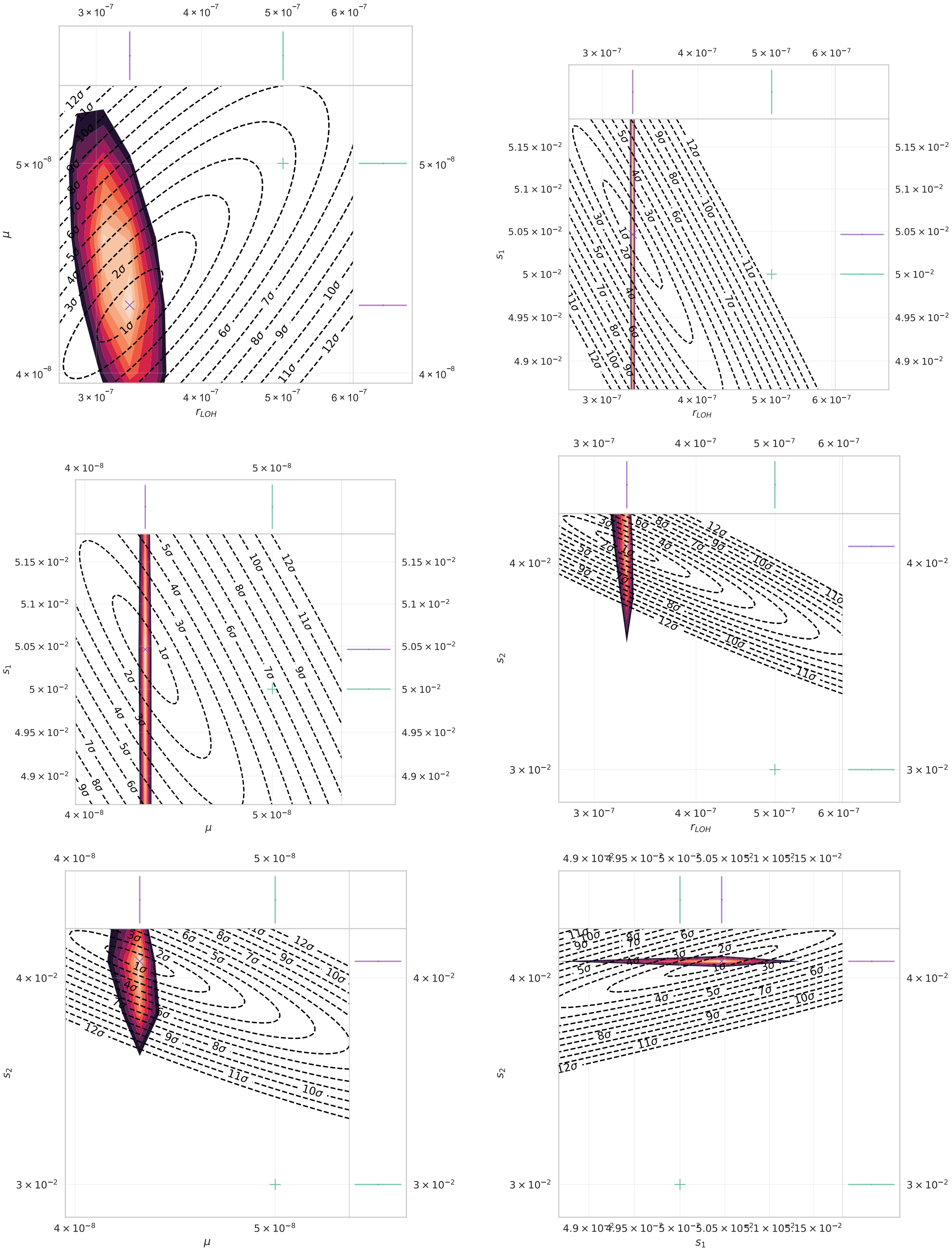

Fig M: Results from the test harness, showing the parameter values for the ground truth (green +), best guess (purple x); a cross section of the likelihood function as a heat map (light orange to black), with likelihood levels that correspond to usual confidence intervals; confidence intervals derived from the Fisher information matrix (dashed ellipses), and a point cloud representing resampled best guesses (grey circles). Top left: μ and r_{LOH}; top right: r_{LOH} and s_1; centre left: μ and s_1; centre right: r_{LOH} and s_2; bottom left: μ and s_2; bottom right: s_1 and s_2. Sample size $N = 10000$.
