# Supplementary material for: A fast numerical integration scheme for clonal expansion processes on graphs
Source: PLoS Comput Biol. 2026 Aug 4;22(8):e1012784. doi: 10.1371/journal.pcbi.1012784 (PMC13436781; doi:10.1371/journal.pcbi.1012784)
Supplement: Supplementary file 2 [file pcbi.1012784.s010.pdf]

The paper "*A Fast Numerical Integration Scheme for Clonal Expansion Processes on Graphs*" by Paterson et al. introduces a significant advancement in modelling cancer progression through the development of a fast numerical integration scheme. While stochastic simulations like Gillespie's algorithm provide exact solutions, they are computationally expensive for complex systems. The authors propose a deterministic approach based on the Kolmogorov forward equations, which enables the direct computation of survival probabilities without the need for stochastic sampling. Their method is computationally efficient, improves parameter inference for mutation rates and selection advantages, and extends beyond linear models to arbitrarily directed graphs. This approach enhances cancer risk modelling and offers broader applications in biological and chemical systems, providing a powerful alternative to traditional stochastic methods for large-scale or graph-based scenario problems.

Below, I have outlined some minor comments for the authors' consideration:

Comments

- In Section 2, where the authors discuss methods for estimating $S_f(t)$ and highlight the assumption of constant rate coefficients:
    - How does the assumption of constant rate coefficients ($\alpha_j, \beta_j, \mu_{jk}, \kappa_j$) impact the biological realism of the model? Specifically, does this assumption adequately capture age-dependent changes in mutation rates or other biological processes?

- Section 2.5: How well does the proposed maximum likelihood estimation method perform when applied to more complex or less ideal datasets, such as those with missing data, small sample sizes, or increased variability in age and tumour type distributions?

- In section 3.1: How do the scaling of errors and runtime with step size Δt in the fast forward method impact its practical applicability for different levels of error tolerance (ε) compared to Gillespie's algorithm, particularly regarding computational efficiency for large-scale models?

- Section 3.2: What factors contribute to the divergence between the Kaplan-Meier estimators from Gillespie simulations and the S_k-curves from the fast-forward method at late ages?

- Figure 5: How does the significantly faster scaling ($T \sim \varepsilon^{-1/2}$) impact its practical usability for large-scale simulations compared to the slower scaling ($T \sim \varepsilon^{-2}$) of Gillespie's algorithm and τ-leaping, particularly for achieving high accuracy?
