# Supplementary material for: A fast numerical integration scheme for clonal expansion processes on graphs
Source: PLoS Comput Biol. 2026 Aug 4;22(8):e1012784. doi: 10.1371/journal.pcbi.1012784 (PMC13436781; doi:10.1371/journal.pcbi.1012784)
Supplement: Supplementary file 3 [file pcbi.1012784.s011.pdf]

I referee this paper with the criteria in mind that are listed on PLOS Computational Biology's web page: Originality, Innovation, High importance to researchers in the field, Significant biological and/or methodological insight, Rigorous methodology, Substantial evidence for its conclusions.

Overall, I believe the work could fulfil these criteria, but I cannot be certain at this point. I have two main concerns, which I will address in turn. One is regarding the method, and the second concerns the presentation in the manuscript. Considerable changes are needed in the manuscript to address these.

<u>Method:</u>
I could not understand the true novelty of the method. If I get this right, then the authors start by carrying out a generating-function analysis of the master equation (Kolmogorov forward eq). This leads to Eqs. (14)-(16). This system needs to be solved numerically, and the authors discuss several ways of doing this (Secs 2.3 and 2.4). If I am not mistaken, the authors then claim that this approach is much faster than simulating the original stochastic process via the Gillespie algorithm.

What I am not convinced of is that this is the right comparison to make. Eqs. (14)-(16) are still a high-dimensional system of coupled ordinary differential equations, and in fact of a similar dimensionality than the Kolmogorov forward eq. (2), which can also be seen as a system of ODEs. So what I do not understand is in what way it is easier to solve this system numerically, than the original master/Kolmogorov forward equation (2). The solution of that equation contains full information about the statistics of the system, and therefore about any quantity that one might be interested in.

One could attempt to solve (2) numerically, e.g. using Euler-forward, RK4 or some other method, or perhaps one could use the methods discussed in Sections 2.3 and 2.4 for that equation (I do not know if this is possible). In any event, my main question is: why is solving (14)-(16) numerically more advantageous than solving (2)? To me, this is the crucial point, not the comparison against Gillespie.

<u>Presentation:</u>
I found it rather difficult to read the paper. The main problem is that the authors mix the general narrative with mathematical and technical details. This makes it hard to identify the main ideas in a first reading. The flow between the sections is rather unclear.

<u>Examples (this is not an exhaustive list):</u>
- The authors provide technical details of birth-death processes in the second paragraph of the introduction, when the general ideas of the paper have not yet been explained. The rest of the introduction is then also quite technical, and I had trouble to understand what the authors meant by "linear graph", and why Eq (1) was necessary in the introduction.

-Sharp jump from Sec 2.1 to 2.2, with zero guidance to the reader.

- The derivation of (14)-(16) is not immediately important to the reader (but should be provided in an appendix/supplement). Instead, I would present the final result, (14)-(16) and

explain the structure of these equations. I was not sure if all quantities in these equations were properly defined. It would also be helpful to give the reader some orientation as to what these quantities mean biologically. The “Proof” on page 6 belongs in the supplement/appendix.

- Secs 2.3 and 2.4 are also very detailed. Not all readers will want to work through all these technical details, they will want to know what the main ideas of these methods are. So the authors need to find a way to synthesise this in the main text, and to move details to an appendix/supplement.
I would also liked to have known more about the novelty of the methods in Secs 2.3 and 2.4. I.e., are these standard methods? What improvements have the authors made? What exactly is the novel contribution here?

-Sec 3 is less technical, but still I think it could be condensed or perhaps be structured in a different way to make things more accessible to the reader. Sec 3.1 for example looks fairly technical – do all readers really need this information, or is this better placed in an appendix? Is it really important that the reader knows the name of the computer cluster in Manchester? To me this looks like meaningless information. Sec 3.3 contains sentences such as “sample sizes … were evenly spaced on a logarithmic scale”. This is nice, but is that really key information that the reader needs?

- I could not tell from the conclusions what the key new idea is here. It would be good if the authors could state this, in a non-technical manner. The statement: “In short we have established that the Kolmorogov forward equations provide a viable approach for estimating hazard functions and likelihoods” is not a characterisation of the main achievements. The Kolmogorov forward equations contain full information about the statistics of the system, and given this, the statement as it is made is obvious and not a result of this paper. I also believe, that this is not what the authors mean – this is why I am asking for a precise non-technical summary of the main achievements.

All in all, significant parts of the manuscript read more like a an internal technical report than a paper. The text needs to be de-cluttered. More synthesis is needed, and the flow and story line needs to be improved, always with the reader in mind. The main purpose of the paper should not be to document what you have done, but rather to communicate this to the readers. The reader needs some guidance as to what is going on, how the different (sub) sections relate to one another, what the main ideas are, what is important and what is not. It is important to keep in mind that some of the intended readers will be experts in simulation methods, but that there will also be non-expert readers, who will only want to understand the main ideas, but not the technical details.

<u>Minor other comments:</u>
- SNP, LOH etc do not seem to be explained in Fig 2.
- In the conclusions the authors say that “This computation of likelihood functions was not previously feasible.” That seems to be a rather strong statement. Has this been stated as an open problem in the literature? I assume that what the authors mean “not feasible within sensible computing time” or something to this effect. Also, the statement is probably not true for all models in general.

- I did not understand the connection to neural networks in the conclusions. I did not understand the relevance of any similarity of the survival function to the activation function.

Acceptance Criteria:

From the present manuscript I cannot decide with confidence if the criteria relating to originality, innovation, and high importance are fulfilled. This is because I do not fully understand the exact advances. The work is within remit, and has biological significance in principle, but again I cannot confirm if there is sufficient "methodological insight". The methods seem to be rigorous, but the evidence for the overall claim that this constitutes a superior new approach to compute survival probabilities is not sufficiently transparent.

Given that this may be a communication issue, rather than the actual substance, I look forward to reading an improved manuscript.
