# Supplementary material for: A fast numerical integration scheme for clonal expansion processes on graphs
Source: PLoS Comput Biol. 2026 Aug 4;22(8):e1012784. doi: 10.1371/journal.pcbi.1012784 (PMC13436781; doi:10.1371/journal.pcbi.1012784)
Supplement: Supplementary file 4 [file pcbi.1012784.s012.docx]

Rebuttal to referees

Referee #1:

*The paper “A Fast Numerical Integration Scheme for Clonal Expansion Processes on Graphs” by Paterson et al. introduces a significant advancement in modelling cancer progression through the development of a fast numerical integration scheme. While stochastic simulations like Gillespie’s algorithm provide exact solutions, they are computationally expensive for complex systems. The authors propose a deterministic approach based on the Kolmogorov forward equations, which enables the direct computation of survival probabilities without the need for stochastic sampling. Their method is computationally efficient, improves parameter inference for mutation rates and selection advantages, and extends beyond linear models to arbitrarily directed graphs. This approach enhances cancer risk modelling and offers broader applications in biological and chemical systems, providing a powerful alternative to traditional stochastic methods for large-scale or graph-based scenario problems.*

*Below, I have outlined some minor comments for the authors' consideration:*

We are very grateful to the reviewer for their positive comments on our manuscript; and we are pleased that they found the work and its applications clearly communicated.

- *In Section 2, where the authors discuss methods for estimating S_f_(𝑡) and highlight the*

*assumption of constant rate coefficients:*

- *How does the assumption of constant rate coefficients (α_j_,β_j_,μ_jk_,κ_j_) impact the biological realism of the model? Specifically, does this assumption adequately capture age-dependent changes in mutation rates or other biological processes?*

This is indeed an important limitation. While a completely arbitrary dependence of the parameters on age would introduce problems with identifiability, non-trivial age dependence is known to be necessary to model exposure to mutagens such as a one-time dose of radiation (see R. Meza 2008). Mathematically, these exposures could be modelled as a delta-function or a piecewise constant function. In these cases, the one-pass method we describe could not be used. While we consider a detailed treatment to be beyond the scope of this paper, this is an important line of questioning for future research, and we can make some concrete questions about how to proceed.

Namely, while the method of characteristics as we described it is only appropriate for first-order reaction networks (as far as we know), variable coefficients could be incorporated using a two-pass method, with one variable for the characteristic time coordinate and a second variable for age/cohort. Quinn’s method of 1989 (“Q89”) is one such approach which can include variable coefficients. However, for reasons discussed in the paper, Q89 is asymptotically not faster than Gillespie’s algorithm, scaling as *T ~ O(ε^-2^)*. This would be partly mitigated by using improved Euler integration: substituting instead a higher-order integration scheme like a fourth-order Runge-Kutta (“RK4”) should result a truly fast method that could incorporate variable mutation rates and fitnesses. This is all a purely theoretical “back-of-the-envelope” argument, and we consider an actual set of simulations to evaluate this to be out of scope of the current work. Nonetheless, an algorithm that couples RK4 to Q89 seems like a sensible and natural next step for future work.

Nonetheless, we still feel that our approach here will enable a more detailed analysis of genomic epidemiology data than was previously possible, and we are looking forward to using it on our ongoing projects to study differences in mechanisms of loss of specific tumour suppressor genes. While doing this, this method’s embedded assumption of constant mutation rates should be interpreted as giving a kind of time-averaged mutation rate.

We have edited a few points in the manuscript to reflect this limitation, as well as proposing concrete next steps to address it. In particular, we have expanded and clarified the Discussion to include the above details on the “RK4+Q89” proposal and the note about careful interpretation.

- *Section 2.5: How well does the proposed maximum likelihood estimation method perform when applied to more complex or less ideal datasets, such as those with missing data, small sample sizes, or increased variability in age and tumour type distributions?*

As far as missing data are concerned, the results of the maximum likelihood estimation appeared to be robust to deletions of individual datums, and thus also fairly robust to outliers. This was established by cross-validating the inference procedure with jack-knife resampling.

The smallest sample sizes studied had a sample size of only 10 patients. While there is an unavoidable loss of statistical power for studies at these small sizes, it was still noted that the statistical estimation scheme produced plausible order-of-magnitude estimates within two standard deviations of the ground-truth value, with no prior information. We considered this to be a success, showing that this inference scheme should be useful for most datasets available from longitudinal studies, even for rare diseases. It was important to us during the development of this project that the software should be tested on datasets that are realistically small, which we took to be sample sizes of 10-100.

We have amended the Results section of the manuscript to emphasise the above points.

- *In section 3.1: How do the scaling of errors and runtime with step size Δ𝑡 in the fast forward method impact its practical applicability for different levels of error tolerance (ε) compared to Gillespie’s algorithm, particularly regarding computational efficiency for large-scale models?*

The answer to your question depends on what you mean by “large scale”. If you simply mean more replicates for the Gillespie algorithm for a lower tolerance, then this new method will always be faster.

If instead by “large scale” you mean that the population size is arbitrarily large, then the answer is more interesting: the run-time and accuracy of the new method is independent of the total population size, whereas for Gillespie, since the event rate is proportional to population, the run-time is also known to increase with population, and as the population becomes very large the simulation becomes very slow. This phenomenon should not happen with our new method.

If, finally, by “large scale” you mean merely that the underlying graph is large, with *|V|* nodes, then since the number of operations in the time stepper is proportional to *|V|*, then this method will become slower as *|V|* increases. The Gillespie algorithm has the same scaling.

We would like to thank the referee for asking this question, as we had previously only considered scaling with respect to error threshold *ε* and not these other factors. We have now noted this in the Results section, and added a brief discussion to a new appendix. This is because we restructured the introduction to the Results section following comments by Referee #2, and wanted to avoid making the Results section longer.

- *Section 3.2: What factors contribute to the divergence between the Kaplan-Meier estimators from Gillespie simulations and the 𝑆_k_-curves from the fast-forward method at late ages?*

There was some discussion of this point in the manuscript, which we have now expanded.

There are two factors which contribute to this divergence. One is that the sample sizes are, in all cases, finite, and thus there is some uncertainty in the Kaplan-Meier (“KM”) estimators. This uncertainty increases as fewer data remain: that is, at late ages. Fewer and fewer patients will survive to old age, so the estimator is less trustworthy here. We have remade these plots with 95% confidence intervals for the KM estimators with n=1000 runs to illustrate this.

The second, much more important, factor is that the KM estimator is *non-parametric*. We know from prior theoretical work by G. Luebeck et al. that the exponential tail seen at long ages in this model is a very robust prediction. (Indeed, in some sense it follows from Frobenius’ theorem for stochastic matrices, so should be totally generic.) However, the KM estimator will always appear flat wherever data are very sparse, such as towards the extreme old ages in any finite dataset. The slope of that exponential tail is a parameter: it cannot be detected and captured by a non-parametric estimator. When we have strong *a priori* reasons to expect trends in datasets, we should also expect non-parametric estimators to deviate from these, and thus to be intrinsically biased towards “flatness”.

As well as a summary of the two main regie have restructured this part of the Results to draw attention to our discussion of this deviation, and would like to thank the referee for drawing our attention to this point.

We have also reviewed the manuscript and methods and made a minor correction: technically, we used Aalen-Johansen estimators, not Kaplan-Meier estimators, to account for multiple competing end states *f*. We have corrected this terminological point in the manuscript, although this is a minor technical detail.

- *Figure 5: How does the significantly faster scaling ([...]) impact its practical usability for large-scale simulations compared to the slower scaling ([...]) of Gillespie’s algorithm and τ-leaping, particularly for achieving high accuracy?*

Since this question overlaps significantly with question 3, we would like to refer you to our answer there. We hope that the additional restructuring of the Results section has also clarified our discussion of asymptotic scaling and practicality.

We would like to thank the referee again for her time and positive comments.

Referee #2:

We would like to thank the reviewer for their many constructive comments regarding the clarity and novelty of the manuscript. We have carefully addressed their questions by clarifying our language, and placing our work into a clearer historical context, which has also provided a better overarching narrative. In addition, many technical details not essential to the narrative flow have been moved to appendices or deleted, in line with the reviewer’s suggestions.

*I referee this paper with the criteria in mind that are listed on PLOS Computational Biology’s*

*web page: Originality, Innovation, High importance to researchers in the field, Significant*

*biological and/or methodological insight, Rigorous methodology, Substantial evidence for its*

*conclusions.*

*Overall, I believe the work could fulfil these criteria, but I cannot be certain at this point. I*

*have two main concerns, which I will address in turn. One is regarding the method, and the*

*second concerns the presentation in the manuscript. Considerable changes are needed in*

*the manuscript to address these.*

***Method:***

*I could not understand the true novelty of the method. If I get this right, then the authors*

*start by carrying out a generating-function analysis of the master equation (Kolmogorov*

*forward eq). This leads to Eqs. (14)-(16). This system needs to be solved numerically, and*

*the authors discuss several ways of doing this (Secs 2.3 and 2.4). If I am not mistaken, the*

*authors then claim that this approach is much faster than simulating the original stochastic*

*process via the Gillespie algorithm.*

While numerical solutions to Kolmogorov forward equations have been studied before, the true novelty of our method is that it is asymptotically much faster than previous methods. Exact solutions (i.e. analytical solutions) are extremely rare, and as such any given numerical solution for one of the probabilities can only be accurate to within some error tolerance, which we denote *ε*. For any given method, we much choose a tolerance and ask how long the method will take to produce an answer. As *ε* decreases, the processing time will increase. At sufficiently small *ε*, our new method is much faster than other known methods for this particular class of problem. We have added a section to the introduction to clarify this, and moved some other technical details to the appendix.

Specific discussions that deal with solving Kolmogorov equations in detail include both the textbook by Winkelmann & Schütte and some reviews by Ramón Grima. We have now cited these in the manuscript.

*One could attempt to solve (2) numerically, e.g. using Euler-forward, RK4 or some other*

*method, or perhaps one could use the methods discussed in Sections 2.3 and 2.4 for that*

*equation (I do not know if this is possible). In any event, my main question is: why is solving*

*(14)-(16) numerically more advantageous than solving (2)? To me, this is the crucial point,*

*not the comparison against Gillespie.*

It is true that the PDE form is also high dimensional, like the original ODE hierarchy. Since the ODE hierarchy is infinite dimensional, and the PDE is equivalent, they must both be infinite dimensional in some sense. To solve the system in either form, the system must be simplified or truncated. Different ways of truncating the system are discussed by Winkelmann and Schutte’s book and by Ramon Grima’s review. Truncating the infinite hierarchy of ODEs is analogous to approximating the derivative operator in the PDE form with a finite difference approximation.

The first key advantage of (14)-(16) lies in the method of characteristics. This means the dimensionality of the system can be radically truncated. Instead of a high (infinite!) dimensional system, we can solve a low dimensional system instead. While it is possible to solve this low-dimensional system numerically, and do so faster than Gillespie, previous work was not *asymptotically* faster. At any chosen error tolerance, the solution would be faster only by a constant multiple.

Once the system is re-expressed in this form, choosing an efficient numerical integration scheme allows us to solve the system not just faster, but *asymptotically* much faster than any previous method. That is, for sufficiently small error tolerances, not only can we find a solution much faster than Gillespie while being just as accurate, but the improvement actually increases as the error tolerance is lowered.

We have amended the introduction to emphasise this, as it is the main idea that holds the narrative of the rest of the work together.

*Presentation:*

*I found it rather difficult to read the paper. The main problem is that the authors mix the*

*general narrative with mathematical and technical details. This makes it hard to identify the*

*main ideas in a first reading. The flow between the sections is rather unclear.*

*Examples (this is not an exhaustive list):*

*- The authors provide technical details of birth-death processes in the second paragraph of*

*the introduction, when the general ideas of the paper have not yet been explained. The rest*

*of the introduction is then also quite technical, and I had trouble to understand what the*

*authors meant by “linear graph”, and why Eq (1) was necessary in the introduction.*

We have rewritten the second paragraph of the introduction to address this, and also corrected our imprecise language. All mentions of “linear graph” have been replaced with “path graph”, to reflect the graph-theoretic literature. For example, RJ Trudeau 1993. Equation (1) and the technical details of how Sf(t) should be defined for general MSCE models has been moved to a new appendix.

*-Sharp jump from Sec 2.1 to 2.2, with zero guidance to the reader.*

We have included a comment so that this flows better.

*- The derivation of (14)-(16) is not immediately important to the reader (but should be*

*provided in an appendix/supplement). Instead, I would present the final result, (14)-(16) andexplain the structure of these equations. I was not sure if all quantities in these equations*

*were properly defined. It would also be helpful to give the reader some orientation as to*

*what these quantities mean biologically. The “Proof” on page 6 belongs in the*

*supplement/appendix.*

In line with the reviewer’s suggestion we have moved the technical details, along with some other definitions in the introduction, into a new appendix.

*- Secs 2.3 and 2.4 are also very detailed. Not all readers will want to work through all these*

*technical details, they will want to know what the main ideas of these methods are. So the*

*authors need to find a way to synthesise this in the main text, and to move details to an*

*appendix/supplement.*

*I would also liked to have known more about the novelty of the methods in Secs 2.3 and 2.4.*

*I.e., are these standard methods? What improvements have the authors made? What*

*exactly is the novel contribution here?*

The technical details of Quinn’s method have been moved to an appendix, replaced in the main text with a high-level overview of the gist of these methods, and the reader has been pointed to other relevant literature on these methods. We have additionally restructured this section to draw attention to our original results, as well as rewriting the Introduction and Discussion.

*-Sec 3 is less technical, but still I think it could be condensed or perhaps be structured in a*

*different way to make things more accessible to the reader. Sec 3.1 for example looks fairly*

*technical – do all readers really need this information, or is this better placed in an*

*appendix? Is it really important that the reader knows the name of the computer cluster in*

*Manchester? To me this looks like meaningless information. Sec 3.3 contains sentences such*

*as “sample sizes … were evenly spaced on a logarithmic scale”. This is nice, but is that really key information that the reader needs?*

We have condensed this section and would like to thank the referee again for their comments. We agree that the name of the computer cluster is irrelevant, and included details about it only because hardware can intrinsically affect run-time. Nonetheless, we moved this sentence out of the main text and into the relevant figure caption.

*- I could not tell from the conclusions what the key new idea is here. It would be good if the*

*authors could state this, in a non-technical manner. The statement: “In short we have*

*established that the Kolmorogov forward equations provide a viable approach for*

*estimating hazard functions and likelihoods” is not a characterisation of the main*

*achievements. The Kolmogorov forward equations contain full information about the*

*statistics of the system, and given this, the statement as it is made is obvious and not a*

*result of this paper. I also believe, that this is not what the authors mean – this is why I am*

*asking for a precise non-technical summary of the main achievements.*

This is indeed not what we meant, and we would like to thank the referee for pointing out that this was unclear. We have rephrased this sentence completely and tried to give a clearer picture of our main achievements. Namely, that this is an asymptotically fast method for computing survival probabilities in this class of model (multi-stage clonal expansion processes on graphs): the main advance is that it is asymptotically faster than the state-of-the-art. By asymptotically faster, we mean the improvement in convergence *accelerates* as the desired precision increases.

We have clarified this point at several times in the text, as well as adding a non-technical summary of what we see as our main achievements at the end of the discussion.

*All in all, significant parts of the manuscript read more like a an internal technical report*

*than a paper. The text needs to be de-cluttered. More synthesis is needed, and the flow*

*and story line needs to be improved, always with the reader in mind. The main purpose of*

*the paper should not be to document what you have done, but rather to communicate this*

*to the readers. The reader needs some guidance as to what is going on, how the different*

*(sub) sections relate to one another, what the main ideas are, what is important and what is*

*not. It is important to keep in mind that some of the intended readers will be experts in*

*simulation methods, but that there will also be non-expert readers, who will only want to*

*understand the main ideas, but not the technical details.*

We really appreciate the time that the referee has taken to give such a detailed set of concrete recommendations, as well as their general concerns about the state of the text. We have moved many technical details and redundant discussions to appendices, and focused on rewording the overall narrative to communicate the advance we have made to the general reader and to better place it in context.

Although the referee classified their final suggestions as “minor”, we have addressed them nonetheless.

*Minor other comments:*

*- SNP, LOH etc do not seem to be explained in Fig 2.*

This has been fixed.

*- In the conclusions the authors say that “This computation of likelihood functions was not*

*previously feasible.” That seems to be a rather strong statement. Has this been stated as an*

*open problem in the literature? I assume that what the authors mean “not feasible within*

*sensible computing time” or something to this effect. Also, the statement is probably not*

*true for all models in general.*

This is indeed what we meant. Furthermore, we have clarified what class of models this applies to: MSCE models on directed graphs specifically. There was not previously a computationally feasible method for computing likelihood functions for these methods that could be computed in a sensible time. We have amended the manuscript to reflect this.

We suspect there are other connections to first-order chemical reaction networks, because on paper it appears the two are equivalent. However, we will have to leave a broader discussion of this to other authors as a detailed review of other fields is out of scope for this manuscript.

*- I did not understand the connection to neural networks in the conclusions. I did not*

*understand the relevance of any similarity of the survival function to the activation function.*

Since this was a speculative comment, and the resemblance might have turned out to be superficial, we have deleted this passage.

We would like to thank the referee again for their time and comments. We believe the manuscript has been substantially improved as a result.

*Acceptance Criteria:*

*From the present manuscript I cannot decide with confidence if the criteria relating to*

*originality, innovation, and high importance are fulfilled. This is because I do not fully*

*understand the exact advances. The work is within remit, and has biological significance in*

*principle, but again I cannot confirm if there is sufficient “methodological insight”. The*

*methods seem to be rigorous, but the evidence for the overall claim that this constitutes a*

*superior new approach to compute survival probabilities is not sufficiently transparent.*

*Given that this may be a communication issue, rather than the actual substance, I look*

*forward to reading an improved manuscript.*
