# Supplementary material for: A fast numerical integration scheme for clonal expansion processes on graphs
Source: PLoS Comput Biol. 2026 Aug 4;22(8):e1012784. doi: 10.1371/journal.pcbi.1012784 (PMC13436781; doi:10.1371/journal.pcbi.1012784)
Supplement: Supplementary file 5 [file pcbi.1012784.s013.docx]

Response to Reviewers

Reviewer #1:

*Thank you for carefully and thoughtfully addressing all of my comments and questions. The revisions are clear, well-justified, and responsive. I appreciate the improvements made to the Discussion section and the clarification regarding constant rate assumptions. I have no further concerns.*

We would like to thank the referee for their previous comments.

Reviewer #2:

*The authors have addressed some of my comments, and I am now largely satisfied that there is sufficient novelty here to meet this journal's standards.

However, the presentation in the manuscript is still deficient. If this had been the first submission I would have recommended major revisions, and so I make the same recommendation here.

My main concern is that large parts of the manuscript are lacking a narrative and flow that can be understood by external readers.*

*Broadly I have two concerns:

1) After reading the paper I still do not understand what the key idea is that leads to the apparent breakthrough in computational speed. I understand that the new method is somehow based on generating functions, method of characteristics etc, and of course I could now work through the detailed mathematics in Sections 2.2-2.5. But not every reader will want to do this. I still think that much of this material should be in an appendix by the way, and that the authors should focus on a set of synthesised main ideas.

Related to this, what is missing is a description of the author's idea at a general but informative level. I.e., a little bit more than "generating functions and method of characteristics", but less than 3 pages of dense mathematical text. What is the underlying principle that makes this algorithm different from existing work? How would you explain this idea informally to someone who is familiar with the theory of stochastic processes? A sort of coffee-break-and-at-the-moment-we-dont-have-a-whiteboard kind of explanation, or, if you want, an elevator pitch to a fellow theoretical modeller.*

We appreciate the referees’ comments, and agree that clarity could be improved by adding a section providing some guidance and intuition for why a change in representation should allow for a breakthrough in computational speed. This new section, “Intuition”, should explain in broad terms why our approach based on generating functions *should* work in a non-technical way*.* We hope that this new section also addresses Referee #2’s broader point about motivation, and would like to thank the referee for prompting us to think seriously about this point.

We also appreciate the reviewer’s suggestion to move more of the technical details into appendices, but have decided to keep the derivations in main text in order to allow readers to engage more deeply with the substance of the manuscript. This has already proved extremely useful to us, since several other scientists have already written in with questions or comments about specific equations, one of which required some re-runs to validate our conclusions. This would have been much less likely if these details were only present in the supplemental material.

*- I found the first two sentences in the author summary misleading: "We are studying the relationship between age, genetics, and cancer risk ... hospital per year.| I think a more accurate summary is that you are developing an algorithm to solve a set of simple stochastic multi-stage models faster. The connection to cancer is remote a this stage, or at least not sufficient to make such a big claim in an opening sentence.*

We would like to thank the referee for this comment: we agree, and these sentences have been changed.

*- page 5: The authors mention "two final nodes f and f'", but I don't understand where this comes from? I thought they are computing S_f for any fixed f? (first paragraph on p. 3). Are they computing the probability of not having reached a fixed node f at time t, or the probability of not having reached any node in a given set by time t? Why two nodes and not a general set? I am a bit lost here.*

This language has been corrected to refer to “sets” instead of pairs, which were a special case.

*- The authors use N both to denote population sizes and number of time steps. I would avoid this.*

This is a good point, this has been corrected. We now use “*R*” for “runs”.

*- Sec 3.3, first sentence "to generate simulated clinical studies". In what way are these simulated clinical studies? Wouldn't it more accurate to say you are generating samples from a simple multi-stage model with unknown parameters and then use your method to estimate these parameters.*

This is an accurate comment and we have rephrased this completely.

Additional revisions:

Shortly after receiving the referees’ reviews, in July 2025 we were also contacted by several other scientists in the field who had studied the preprint and wanted to implement the library. We were surprised by this level of interest. One of these pre-print reviewers noted that one of one of our definitions in the latter section of this manuscript was missing a prefactor: this error had previously been missed by all authors and both referees.

In the course of these revisions, we have had to very carefully chase through the effects of this correction to gauge how our conclusions may have been affected: luckily, it is a small perturbation that does not affect our results qualitatively, and has actually served to strengthen our original conclusions about identifiability of the tumour suppressor test case. All of the figures from this second section have nonetheless been recreated.

The scientist in question is now credited in the acknowledgements alongside all others who volunteered comments on the preprint, and all resulting changes have been highlighted in the revised version.
