# Supplementary material for: A fast numerical integration scheme for clonal expansion processes on graphs
Source: PLoS Comput Biol. 2026 Aug 4;22(8):e1012784. doi: 10.1371/journal.pcbi.1012784 (PMC13436781; doi:10.1371/journal.pcbi.1012784)
Supplement: Supplementary file 6 [file pcbi.1012784.s014.docx]

Response to Reviewers

Reviewer #2:

We would like to thank the reviewer for their definite and constructive criticisms. We are very pleased that the reviewer feels our manuscript has been improved, and we are grateful for the opportunity to improve it further.

*Reviewer #2: As I stated before, I have no doubt that there is sufficient substance in the paper for it to be publishable. The gain in run time in Fig 5 is impressive. I note my comment 7) below though - the novelty of the idea underpinning this work needs to be stated with more precision.

However, the presentation in the manuscript is still not very clear and I think it is in the authors' own interest to work on this so as to maximise the uptake of their work.

1) page 3: The coefficients alpha_j, beta_j, mu_{jk} and kappa_j seem to simply appear in the text (second paragraph). As far as I can tell they hadn't been introduced previously, so the reader is left to guess what these coefficients represent.*

This has been amended: the coefficients are now defined explicitly in the text. *2) page 4: I appreciate the new section 2.1, but unfortunately the text is not very clear. I do not understand what it means "to solve for all the terms (1) directly". Solve for a term? As a consequence I do not fully understand the first paragraph of Sec 2.1.*

We are grateful to the referee for pointing this out: we have rephrased this section to make our meaning more clear, so that the approach is now to try to “directly construct the probability distribution *P(t, N)* that solves *(1)*.”. *3) Following on from this, in the second paragraph of Sec 2.1 the authors mention a representation of P(t,N) as a smooth travelling wave. I was not able to relate the later text to this statement. I understand the authors effectively switch to Fourier space later, but I find it difficult to identify a "smooth travelling" wave. I think it would be good if the authors could elaborate.*

By this phrase, we meant to give an intuitive summary of the rest of our approach -- the passage to *Ψ* as a differentiable function, which we solve by the method of characteristics – emphasising that *Ψ* is differentiable (“smooth”) and solutions can be constructed by tracing “rays” (characteristics), like other travelling waves. We are pleased to have the opportunity to elaborate on this point, and have expanded the relevant passage. *4) page 4, algorithm at the bottom, a_j(N) seems undefined*

Thank you, we have added an explanation of this rate term with two references. *5) (minor comment) page 5, second line, the value Y=0 needs to be excluded I think*

You are quite correct, thank you. We have corrected this in the text.

*6) (minor comment) page 6, I noticed that Winkelman and Schütte are mentioned only by surname, but Grima with full name. This seems inconsistent.*

We have corrected this.

***7) (crucial comment): The fundamental step of the work is still not clear.*** *I understand that the master equation (1) is a set of infinitely many coupled ODEs. The authors then go to Fourier space by writing down (4) and finding (11). So far, so good. This is the standard moment generating function representation of a master equation.*

***In the last paragraph of Sec 2.3 the authors then mention that (10) has a particular symmetry, and that this allows for a reduction of the dimensionality of the problem, via the method of characteristics. But what is this symmetry?*** *The method of characteristics is quite standard, and so I don't really understand what the key idea is here, and how much of this is applying previous knowledge (method of characteristics) and how much is genuinely new.

What is it about this system that allows for the reduction of the dimensionality? What is the symmetry the authors are referring to? In what way is this feature not present in other systems, and in what way is the reduction of dimensionality via the method of characteristics new? I thought it was quite standard to use the method of characteristics to reduce the PDE coming from a generating function representation of a master equation to an ODE, or to a set of ODEs if there are multiple species of particle. (I have not done a detailed search now, but I am pretty sure this can be found in textbooks or lecture notes). So what exactly is the new idea here?

I am not saying there has to be a key new idea, but the authors a) have to explain more clearly what "symmetry" they are talking about, b) what principle/property of this particular system allows them to carry out the reduction of the initial master equation to a finite set of ODEs (is this the fact that each reaction only changes any of the N_j bu plus/minus 1?), and c) represent with more precision the degree of novelty of these ideas.*

The key property is hyperbolicity. We have completely rewritten this paragraph so that our language is more precise, and would like to thank the referee for their attention. We know that all the reactions being first-order is sufficient for the system to be linear and hyperbolic, and we do not think that it is necessary for each reaction to only change any *N_j* by +- 1: while we aren’t totally sure of the *necessary* conditions for hyperbolicity, it might be related the fact that there are a finite number of reactions that can affect any of the populations *j*.

We are grateful to the referee for this comment, which provoked some interesting reflections and discussions. *8) I did not understand the organisation of the text in Sections 2.3 and 2.4 The authors give (correctly I think) Eq (6) in Sec. 2.3. Then on the next page (page 7) they have Theorem I, and then from this the authors seem to derive (15). They point to appendix B for a proof. But (15) is a special case of (6), isn't it? What is the logical flow of the text on pages 5 and 6?*

We organized this text so that the theorem on which the algorithm relies is presented first, and in a typical mathematical style: definitions of all important terms, then the statement of the theorem, then the proof (now in an appendix). Page 5 is underlying definitions before the statement of theorem 1 on page 6. We have edited how these sections are labeled to make this clearer, and would like to thank the referee for their comment. *9) I did not understand the first paragraph on page 7. I agree that solving a finite set of ODEs is more efficient than running Gillespie simulations. But the text suggests that the Gillespie method is somehow based on backward equations, which isn't really true.*

We meant only that the prior work of some coauthors on reduction to Gauss-Konrod quadrature was based on backward equations: we have rewritten this paragraph on page 7 to clarify this (with relevant references). *10) Overall I maintain that a shorter, more concise manuscript would be better suited to convey the main ideas. There is too much "clutter" in the manuscript, the main ideas are either not clear or they get a bit lost. At the moment the reader has to work quite hard to separate the clutter from the actual substance. I think I can say this with confidence for the first parts of the paper (Secs 1 and 2) which I read in quite some detail. I understand the authors have tried to address a similar earlier comment in the new Section 2.1, but unfortunately the text they have added is only moderately useful in this regard.

I admit I have not read Sec 3 with the same level of attention, but my impression is that there the text could be streamlined too, and that non-essential statements and discussion would best be relegated to the appendix.*

We would like to thank the referee for their comments, and we appreciate the referee’s concern that the manuscript is cluttered. We have tried to address this by editing several sections down or deleting redundant phrasing, and have indicated where we have done this in our marked-up copy of the manuscript. We have also followed through Section 3 in a similar spirit.
