# Supplementary material for: A fast numerical integration scheme for clonal expansion processes on graphs
Source: PLoS Comput Biol. 2026 Aug 4;22(8):e1012784. doi: 10.1371/journal.pcbi.1012784 (PMC13436781; doi:10.1371/journal.pcbi.1012784)
Supplement: Peer review history

# Peer review: Decision Letter 0

**DOI:** 10.1371/journal.pcbi.1012784.r001

**Authors:** Philipp Martin Altrock, Benjamin M. Althouse

25 Feb 2025

PCOMPBIOL-D-25-00040

A fast numerical integration scheme for clonal expansion processes on graphs

PLOS Computational Biology

Dear Dr. Paterson,

Thank you for submitting your manuscript to PLOS Computational Biology. After careful consideration, we feel that it has merit but does not fully meet PLOS Computational Biology's publication criteria as it currently stands. Therefore, we invite you to submit a revised version of the manuscript that addresses the points raised during the review process.

Please submit your revised manuscript within 60 days Apr 27 2025 11:59PM. If you will need more time than this to complete your revisions, please reply to this message or contact the journal office at ploscompbiol@plos.org. When you're ready to submit your revision, log on to https://www.editorialmanager.com/pcompbiol/ and select the 'Submissions Needing Revision' folder to locate your manuscript file.

Please include the following items when submitting your revised manuscript:

* A rebuttal letter that responds to each point raised by the editor and reviewer(s). You should upload this letter as a separate file labeled 'Response to Reviewers'. This file does not need to include responses to formatting updates and technical items listed in the 'Journal Requirements' section below.

* A marked-up copy of your manuscript that highlights changes made to the original version. You should upload this as a separate file labeled 'Revised Manuscript with Track Changes'.

* An unmarked version of your revised paper without tracked changes. You should upload this as a separate file labeled 'Manuscript'.

If you would like to make changes to your financial disclosure, competing interests statement, or data availability statement, please make these updates within the submission form at the time of resubmission. Guidelines for resubmitting your figure files are available below the reviewer comments at the end of this letter

We look forward to receiving your revised manuscript.

Kind regards,

Philipp Martin Altrock, Ph.D.

Academic Editor

PLOS Computational Biology

Benjamin Althouse

Section Editor

PLOS Computational Biology

**Journal Requirements:**

1) Please ensure that the CRediT author contributions listed for every co-author are completed accurately and in full.

At this stage, the following Authors/Authors require contributions: Chay Giles Blair Paterson, Miaomiao Gao, Joshua Hellier, Georg Luebeck, David C Wedge, and Ivana Bozic. Please ensure that the full contributions of each author are acknowledged in the "Add/Edit/Remove Authors" section of our submission form.

The list of CRediT author contributions may be found here: https://journals.plos.org/ploscompbiol/s/authorship#loc-author-contributions

2) We ask that a manuscript source file is provided at Revision. Please upload your manuscript file as a .doc, .docx, .rtf or .tex. If you are providing a .tex file, please upload it under the item type u2018LaTeX Source Fileu2019 and leave your .pdf version as the item type u2018Manuscriptu2019.

3) Please provide an Author Summary. This should appear in your manuscript between the Abstract (if applicable) and the Introduction, and should be 150-200 words long. The aim should be to make your findings accessible to a wide audience that includes both scientists and non-scientists. Sample summaries can be found on our website under Submission Guidelines:

https://journals.plos.org/ploscompbiol/s/submission-guidelines#loc-parts-of-a-submission

4) Please upload all main figures as separate Figure files in .tif or .eps format. For more information about how to convert and format your figure files please see our guidelines:

https://journals.plos.org/ploscompbiol/s/figures

5) Please ensure that all Figure files have corresponding citations and legends within the manuscript. Currently, Figure 4 in your submission file inventory does not have an in-text citation. If the figure is no longer to be included as part of the submission, please remove it from the file inventory.

6) We have noticed that you have uploaded Supporting Information files, but you have not included a complete list of legends. Please add a full list of legends for your Supporting Information files after the references list.

7) We notice that your supplementary Figures, and information (Appendices) are included in the manuscript file. Please remove them and upload them with the file type 'Supporting Information'. Please ensure that each Supporting Information file has a legend listed in the manuscript after the references list.

8) Please include a completed 'Competing Interests' statement in the text box when submitting your production task, including any COIs declared by your co-authors, written in full sentences.

If you have no competing interests to declare, please state "The authors have declared that no competing interests exist". You may also provide an updated statement via email.

9) Thank you for stating that "This reference implementation, called "libffwd", will be made available as an open-source repository at the first author's GitHub:https://github.com/chaypaterson/." Please provide us with a direct link to access the dataset.

**Reviewers' comments:**

Reviewer's Responses to Questions

**Comments to the Authors:**

**Please note that two reviews are uploaded as attachments.**

Reviewer #1: The full review has been uploaded as an attachment.

Reviewer #2: See attachment

**********

**Have the authors made all data and (if applicable) computational code underlying the findings in their manuscript fully available?**

The PLOS Data policy requires authors to make all data and code underlying the findings described in their manuscript fully available without restriction, with rare exception (please refer to the Data Availability Statement in the manuscript PDF file). The data and code should be provided as part of the manuscript or its supporting information, or deposited to a public repository. For example, in addition to summary statistics, the data points behind means, medians and variance measures should be available. If there are restrictions on publicly sharing data or code —e.g. participant privacy or use of data from a third party—those must be specified.

Reviewer #1: Yes

Reviewer #2: None

**********

PLOS authors have the option to publish the peer review history of their article (what does this mean?). If published, this will include your full peer review and any attached files.

If you choose “no”, your identity will remain anonymous but your review may still be made public.

**Do you want your identity to be public for this peer review?**  For information about this choice, including consent withdrawal, please see our Privacy Policy.

Reviewer #1: **Yes:**  suzan Farhang-Sardroodi

Reviewer #2: No

[NOTE: If reviewer comments were submitted as an attachment file, they will be attached to this email and accessible via the submission site. Please log into your account, locate the manuscript record, and check for the action link "View Attachments". If this link does not appear, there are no attachment files.]

**Figure resubmission:**

While revising your submission, please upload your figure files to the Preflight Analysis and Conversion Engine (PACE) digital diagnostic tool, https://pacev2.apexcovantage.com/. PACE helps ensure that figures meet PLOS requirements. To use PACE, you must first register as a user. Registration is free. Then, login and navigate to the UPLOAD tab, where you will find detailed instructions on how to use the tool. If you encounter any issues or have any questions when using PACE, please email PLOS at figures@plos.org. Please note that Supporting Information files do not need this step. If there are other versions of figure files still present in your submission file inventory at resubmission, please replace them with the PACE-processed versions.

**Reproducibility:**

To enhance the reproducibility of your results, we recommend that authors of applicable studies deposit laboratory protocols in protocols.io, where a protocol can be assigned its own identifier (DOI) such that it can be cited independently in the future. Additionally, PLOS ONE offers an option to publish peer-reviewed clinical study protocols. Read more information on sharing protocols at https://plos.org/protocols?utm_medium=editorial-email&utm_source=authorletters&utm_campaign=protocols

Attachment Submitted filename: Comments,PLOS Computational Biology,2025.pdf

Attachment Submitted filename: PLOS-CB-D-25-00040_referee_report.pdf

# Author comment: Author response to Decision Letter 1

**DOI:** 10.1371/journal.pcbi.1012784.r002

28 Apr 2025

Attachment Submitted filename: Response to Reviewers.docx

# Peer review: Decision Letter 1

**DOI:** 10.1371/journal.pcbi.1012784.r003

**Authors:** Benjamin M. Althouse

15 Jul 2025

PCOMPBIOL-D-25-00040R1

A fast numerical integration scheme for clonal expansion processes on graphs

PLOS Computational Biology

Dear Dr. Paterson,

Thank you for submitting your manuscript to PLOS Computational Biology. After careful consideration, we feel that it has merit but does not fully meet PLOS Computational Biology's publication criteria as it currently stands. Therefore, we invite you to submit a revised version of the manuscript that addresses the points raised during the review process.

Please submit your revised manuscript within 60 days Sep 09 2025 11:59PM. If you will need more time than this to complete your revisions, please reply to this message or contact the journal office at ploscompbiol@plos.org. When you're ready to submit your revision, log on to https://www.editorialmanager.com/pcompbiol/ and select the 'Submissions Needing Revision' folder to locate your manuscript file.

Please include the following items when submitting your revised manuscript:

* A rebuttal letter that responds to each point raised by the editor and reviewer(s). You should upload this letter as a separate file labeled 'Response to Reviewers'. This file does not need to include responses to formatting updates and technical items listed in the 'Journal Requirements' section below.

* A marked-up copy of your manuscript that highlights changes made to the original version. You should upload this as a separate file labeled 'Revised Manuscript with Track Changes'.

* An unmarked version of your revised paper without tracked changes. You should upload this as a separate file labeled 'Manuscript'.

If you would like to make changes to your financial disclosure, competing interests statement, or data availability statement, please make these updates within the submission form at the time of resubmission. Guidelines for resubmitting your figure files are available below the reviewer comments at the end of this letter

We look forward to receiving your revised manuscript.

Kind regards,

Benjamin Althouse

Section Editor

PLOS Computational Biology

**Journal Requirements:**

1)  Please cite and label the supplementary figures (cited as Figures 12-15 in the manuscript) as "S1 Figure", S2 Figure" and so forth.

2) Please upload a copy of Figure 11 in the online submission form as a separate Figure file in .tif or .eps format. If it is meant to be a table, please cite and label it as Table 1 noting that tables should not be uploaded as individual files.

**Reviewers' comments:**

Reviewer's Responses to Questions

Reviewer #1: Thank you for carefully and thoughtfully addressing all of my comments and questions. The revisions are clear, well-justified, and responsive. I appreciate the improvements made to the Discussion section and the clarification regarding constant rate assumptions. I have no further concerns.

Reviewer #2: The authors have addressed some of my comments, and I am now largely satisfied that there is sufficient novelty here to meet this journal's standards.

However, the presentation in the manuscript is still deficient. If this had been the first submission I would have recommended major revisions, and so I make the same recommendation here.

My main concern is that large parts of the manuscript are lacking a narrative and flow that can be understood by external readers.

Broadly I have two concerns:

1) After reading the paper I still do not understand what the key idea is that leads to the apparent breakthrough in computational speed. I understand that the new method is somehow based on generating functions, method of characteristics etc, and of course I could now work through the detailed mathematics in Sections 2.2-2.5. But not every reader will want to do this. I still think that much of this material should be in an appendix by the way, and that the authors should focus on a set of synthesised main ideas.

Related to this, what is missing is a description of the author's idea at a general but informative level. I.e., a little bit more than "generating functions and method of characteristics", but less than 3 pages of dense mathematical text. What is the underlying principle that makes this algorithm different from existing work? How would you explain this idea informally to someone who is familiar with the theory of stochastic processes? A sort of coffee-break-and-at-the-moment-we-dont-have-a-whiteboard kind of explanation, or, if you want, an elevator pitch to a fellow theoretical modeller.

I've read this work several times now, but I still would not be able to summarise what exactly the advance is, other than "a faster method somehow to do with using generating functions".

2) The authors are not doing enough to guide the reader through their work. I have already commented on the more theoretical parts in Sec 2, but the problem continues in the later parts.

Some of the figures are mentioned only very briefly, but little guidance is given to reader as to the interpretation of the results. Sometimes they do not seem to be mentioned at all at when point would have been helpful. Sometimes figures are referred to as a bulk (p. 19 bottom, "figures 8-10"), but all the authors say is "it can be seen ...". Rather than guiding the reader, the authors leave most of the interpretation work to the reader.

At times the paper is hard to read because too many details are provided (or what is said is said on too much space), and then at other times, guidance is missing. The reader is left to infer what the authors mean. There is at times no clear flow, the reader is sort of left to guess how the different paragraphs hang together. Sometimes the story seemed to jump between different aspects (e.g. p. 19).

I think the paper would be much more pleasant to read if a more empathic style of presentation would be adopted and if the flow and cohesion of the narrative could be improved, with a more thought-out balance between making the manuscript succinct, but also providing all information that is need for the reader to be able to follow without undue effort.

Further comments on specific parts of the manuscript:

- I found the first two sentences in the author summary misleading: "We are studying the relationship between age, genetics, and cancer risk ... hospital per year.| I think a more accurate summary is that you are developing an algorithm to solve a set of simple stochastic multi-stage models faster. The connection to cancer is remote a this stage, or at least not sufficient to make such a big claim in an opening sentence.

- I found the introduction hard to read, in particular the second and third paragraphs on page 2. This is because this is quite technical, but no model has been defined at this stage. I don't think it is helpful to introduce mathematical notation at this stage (S_f, h_f, Psi, Phi...).

- The model definitions on page 3 are not clear. You are not giving the relevant processes (birth, death etc), and it is not clear what the alpha_i, beta_i, etc are. The processes leading to the master equation (1) are not explained. A set of "chemical reactions" and a more pedagogical description of the model would help.

- page 5: The authors mention "two final nodes f and f'", but I don't understand where this comes from? I thought they are computing S_f for any fixed f? (first paragraph on p. 3). Are they computing the probability of not having reached a fixed node f at time t, or the probability of not having reached any node in a given set by time t? Why two nodes and not a general set? I am a bit lost here.

- The authors use N both to denote population sizes and number of time steps. I would avoid this.

- Sec 3.3, first sentence "to generate simulated clinical studies". In what way are these simulated clinical studies? Wouldn't it more accurate to say you are generating samples from a simple multi-stage model with unknown parameters and then use your method to estimate these parameters.

**********

**Have the authors made all data and (if applicable) computational code underlying the findings in their manuscript fully available?**

The PLOS Data policy requires authors to make all data and code underlying the findings described in their manuscript fully available without restriction, with rare exception (please refer to the Data Availability Statement in the manuscript PDF file). The data and code should be provided as part of the manuscript or its supporting information, or deposited to a public repository. For example, in addition to summary statistics, the data points behind means, medians and variance measures should be available. If there are restrictions on publicly sharing data or code —e.g. participant privacy or use of data from a third party—those must be specified.

Reviewer #1: Yes

Reviewer #2: Yes

**********

PLOS authors have the option to publish the peer review history of their article (what does this mean?). If published, this will include your full peer review and any attached files.

If you choose “no”, your identity will remain anonymous but your review may still be made public.

**Do you want your identity to be public for this peer review?**  For information about this choice, including consent withdrawal, please see our Privacy Policy.

Reviewer #1: **Yes:**  Suzan Farhang-Sardroodi, Department of Pharmacology & Toxicology, Temerty Faculty of Medicine, University of Toronto

Reviewer #2: No

[NOTE: If reviewer comments were submitted as an attachment file, they will be attached to this email and accessible via the submission site. Please log into your account, locate the manuscript record, and check for the action link "View Attachments". If this link does not appear, there are no attachment files.]

**Figure resubmission:**

While revising your submission, please upload your figure files to the Preflight Analysis and Conversion Engine (PACE) digital diagnostic tool, https://pacev2.apexcovantage.com/. PACE helps ensure that figures meet PLOS requirements. To use PACE, you must first register as a user. Registration is free. Then, login and navigate to the UPLOAD tab, where you will find detailed instructions on how to use the tool. If you encounter any issues or have any questions when using PACE, please email PLOS at figures@plos.org. Please note that Supporting Information files do not need this step. If there are other versions of figure files still present in your submission file inventory at resubmission, please replace them with the PACE-processed versions.

**Reproducibility:**

To enhance the reproducibility of your results, we recommend that authors of applicable studies deposit laboratory protocols in protocols.io, where a protocol can be assigned its own identifier (DOI) such that it can be cited independently in the future. Additionally, PLOS ONE offers an option to publish peer-reviewed clinical study protocols. Read more information on sharing protocols at https://plos.org/protocols?utm_medium=editorial-email&utm_source=authorletters&utm_campaign=protocols

# Author comment: Author response to Decision Letter 2

**DOI:** 10.1371/journal.pcbi.1012784.r004

19 Jan 2026

Attachment Submitted filename: Response_to_Reviewers_auresp_2.docx

# Peer review: Decision Letter 2

**DOI:** 10.1371/journal.pcbi.1012784.r005

**Authors:** Benjamin M. Althouse

1 Mar 2026

PCOMPBIOL-D-25-00040R2

A fast numerical integration scheme for clonal expansion processes on graphs

PLOS Computational Biology

Dear Dr. Paterson,

Thank you for submitting your manuscript to PLOS Computational Biology. After careful consideration, we feel that it has merit but does not fully meet PLOS Computational Biology's publication criteria as it currently stands. Therefore, we invite you to submit a revised version of the manuscript that addresses the points raised during the review process.

Please submit your revised manuscript by May 01 2026 11:59PM. If you will need more time than this to complete your revisions, please reply to this message or contact the journal office at ploscompbiol@plos.org. When you're ready to submit your revision, log on to https://www.editorialmanager.com/pcompbiol/ and select the 'Submissions Needing Revision' folder to locate your manuscript file.

Please include the following items when submitting your revised manuscript:

* A letter that responds to each point raised by the editor and reviewer(s). You should upload this letter as a separate file labeled 'Response to Reviewers'. This file does not need to include responses to formatting updates and technical items listed in the 'Journal Requirements' section below.

* A marked-up copy of your manuscript that highlights changes made to the original version. You should upload this as a separate file labeled 'Revised Manuscript with Track Changes'.

* An unmarked version of your revised paper without tracked changes. You should upload this as a separate file labeled 'Manuscript'.

If you would like to make changes to your financial disclosure, competing interests statement, or data availability statement, please make these updates within the submission form at the time of resubmission. Guidelines for resubmitting your figure files are available below the reviewer comments at the end of this letter

We look forward to receiving your revised manuscript.

Kind regards,

Benjamin Althouse

Section Editor

PLOS Computational Biology

**Additional Editor Comments :**

The reviewer has outlined some significant points that need addressing.

**********

Note: If the reviewer comments include a recommendation to cite specific previously published works, please review and evaluate these publications to determine whether they are relevant and should be cited. There is no requirement to cite these works unless the editor has indicated otherwise.

**Reviewers' comments:**

Reviewer's Responses to Questions

Reviewer #2: As I stated before, I have no doubt that there is sufficient substance in the paper for it to be publishable. The gain in run time in Fig 5 is impressive. I note my comment 7) below though - the novelty of the idea underpinning this work needs to be stated with more precision.

However, the presentation in the manuscript is still not very clear and I think it is in the authors' own interest to work on this so as to maximise the uptake of their work.

1) page 3: The coefficients alpha_j, beta_j, mu_{jk} and kappa_j seem to simply appear in the text (second paragraph). As far as I can tell they hadn't been introduced previously, so the reader is left to guess what these coefficients represent.

2) page 4: I appreciate the new section 2.1, but unfortunately the text is not very clear. I do not understand what it means "to solve for all the terms (1) directly". Solve for a term? As a consequence I do not fully understand the first paragraph of Sec 2.1.

3) Following on from this, in the second paragraph of Sec 2.1 the authors mention a representation of P(t,N) as a smooth travelling wave. I was not able to relate the later text to this statement. I understand the authors effectively switch to Fourier space later, but I find it difficult to identify a "smooth travelling" wave. I think it would be good if the authors could elaborate.

4) page 4, algorithm at the bottom, a_j(N) seems undefined

5) (minor comment) page 5, second line, the value Y=0 needs to be excluded I think

6) (minor comment) page 6, I noticed that Winkelman and Schütte are mentioned only by surname, but Grima with full name. This seems inconsistent.

7) (crucial comment): The fundamental step of the work is still not clear. I understand that the master equation (1) is a set of infinitely many coupled ODEs. The authors then go to Fourier space by writing down (4) and finding (11). So far, so good. This is the standard moment generating function representation of a master equation.

In the last paragraph of Sec 2.3 the authors then mention that (10) has a particular symmetry, and that this allows for a reduction of the dimensionality of the problem, via the method of characteristics. But what is this symmetry? The method of characteristics is quite standard, and so I don't really understand what the key idea is here, and how much of this is applying previous knowledge (method of characteristics) and how much is genuinely new.

What is it about this system that allows for the reduction of the dimensionality? What is the symmetry the authors are referring to? In what way is this feature not present in other systems, and in what way is the reduction of dimensionality via the method of characteristics new? I thought it was quite standard to use the method of characteristics to reduce the PDE coming from a generating function representation of a master equation to an ODE, or to a set of ODEs if there are multiple species of particle. (I have not done a detailed search now, but I am pretty sure this can be found in textbooks or lecture notes). So what exactly is the new idea here?

I am not saying there has to be a key new idea, but the authors a) have to explain more clearly what "symmetry" they are talking about, b) what principle/property of this particular system allows them to carry out the reduction of the initial master equation to a finite set of ODEs (is this the fact that each reaction only changes any of the N_j bu plus/minus 1?), and c) represent with more precision the degree of novelty of these ideas.

8) I did not understand the organisation of the text in Sections 2.3 and 2.4 The authors give (correctly I think) Eq (6) in Sec. 2.3. Then on the next page (page 7) they have Theorem I, and then from this the authors seem to derive (15). They point to appendix B for a proof. But (15) is a special case of (6), isn't it? What is the logical flow of the text on pages 5 and 6?

9) I did not understand the first paragraph on page 7. I agree that solving a finite set of ODEs is more efficient than running Gillespie simulations. But the text suggests that the Gillespie method is somehow based on backward equations, which isn't really true.

10) Overall I maintain that a shorter, more concise manuscript would be better suited to convey the main ideas. There is too much "clutter" in the manuscript, the main ideas are either not clear or they get a bit lost. At the moment the reader has to work quite hard to separate the clutter from the actual substance. I think I can say this with confidence for the first parts of the paper (Secs 1 and 2) which I read in quite some detail. I understand the authors have tried to address a similar earlier comment in the new Section 2.1, but unfortunately the text they have added is only moderately useful in this regard.

I admit I have not read Sec 3 with the same level of attention, but my impression is that there the text could be streamlined too, and that non-essential statements and discussion would best be relegated to the appendix.

**********

**Have the authors made all data and (if applicable) computational code underlying the findings in their manuscript fully available?**

The PLOS Data policy requires authors to make all data and code underlying the findings described in their manuscript fully available without restriction, with rare exception (please refer to the Data Availability Statement in the manuscript PDF file). The data and code should be provided as part of the manuscript or its supporting information, or deposited to a public repository. For example, in addition to summary statistics, the data points behind means, medians and variance measures should be available. If there are restrictions on publicly sharing data or code —e.g. participant privacy or use of data from a third party—those must be specified.

Reviewer #2: Yes

**********

PLOS authors have the option to publish the peer review history of their article (what does this mean?). If published, this will include your full peer review and any attached files.

If you choose “no”, your identity will remain anonymous but your review may still be made public.

**Do you want your identity to be public for this peer review?**  For information about this choice, including consent withdrawal, please see our Privacy Policy.

Reviewer #2: No

[NOTE: If reviewer comments were submitted as an attachment file, they will be attached to this email and accessible via the submission site. Please log into your account, locate the manuscript record, and check for the action link "View Attachments". If this link does not appear, there are no attachment files.]

**Figure resubmission:**

While revising your submission, we strongly recommend that you use PLOS’s NAAS tool (https://ngplosjournals.pagemajik.ai/artanalysis) to test your figure files. NAAS can convert your figure files to the TIFF file type and meet basic requirements (such as print size, resolution), or provide you with a report on issues that do not meet our requirements and that NAAS cannot fix.

After uploading your figures to PLOS’s NAAS tool - https://ngplosjournals.pagemajik.ai/artanalysis, NAAS will process the files provided and display the results in the "Uploaded Files" section of the page as the processing is complete. If the uploaded figures meet our requirements (or NAAS is able to fix the files to meet our requirements), the figure will be marked as "fixed" above. If NAAS is unable to fix the files, a red "failed" label will appear above. When NAAS has confirmed that the figure files meet our requirements, please download the file via the download option, and include these NAAS processed figure files when submitting your revised manuscript.

**Reproducibility:**

To enhance the reproducibility of your results, we recommend that authors of applicable studies deposit laboratory protocols in protocols.io, where a protocol can be assigned its own identifier (DOI) such that it can be cited independently in the future. Additionally, PLOS ONE offers an option to publish peer-reviewed clinical study protocols. Read more information on sharing protocols at https://plos.org/protocols?utm_medium=editorial-email&utm_source=authorletters&utm_campaign=protocols

# Author comment: Author response to Decision Letter 3

**DOI:** 10.1371/journal.pcbi.1012784.r006

1 May 2026

Attachment Submitted filename: Response to Reviewers-4.docx

# Peer review: Decision Letter 3

**DOI:** 10.1371/journal.pcbi.1012784.r007

**Authors:** Benjamin M. Althouse

23 Jun 2026

Dear Dr Paterson,

We are pleased to inform you that your manuscript 'A fast numerical integration scheme for clonal expansion processes on graphs' has been provisionally accepted for publication in PLOS Computational Biology.

Before your manuscript can be formally accepted you will need to complete some formatting changes, which you will receive in a follow up email. A member of our team will be in touch with a set of requests.

Please note that your manuscript will not be scheduled for publication until you have made the required changes, so a swift response is appreciated.

IMPORTANT: The editorial review process is now complete. PLOS will only permit corrections to spelling, formatting or significant scientific errors from this point onwards. Requests for major changes, or any which affect the scientific understanding of your work, will cause delays to the publication date of your manuscript.

Should you, your institution's press office or the journal office choose to press release your paper, you will automatically be opted out of early publication. We ask that you notify us now if you or your institution is planning to press release the article. All press must be co-ordinated with PLOS.

Thank you again for supporting Open Access publishing; we are looking forward to publishing your work in PLOS Computational Biology.

Best regards,

Benjamin M. Althouse

Section Editor

PLOS Computational Biology

Benjamin Althouse

Section Editor

PLOS Computational Biology

***********************************************************

Reviewer's Responses to Questions

**Comments to the Authors: <br/>Please note here if the review is uploaded as an attachment.**

Reviewer #2: The authors have further improved the manuscript. I have additional questions, but I think it would be unreasonable for me to ask for further revisions. Instead I communicate these queries to the Editor, and defer to their judgement at this stage.

**********

**Have the authors made all data and (if applicable) computational code underlying the findings in their manuscript fully available?**

The PLOS Data policy requires authors to make all data and code underlying the findings described in their manuscript fully available without restriction, with rare exception (please refer to the Data Availability Statement in the manuscript PDF file). The data and code should be provided as part of the manuscript or its supporting information, or deposited to a public repository. For example, in addition to summary statistics, the data points behind means, medians and variance measures should be available. If there are restrictions on publicly sharing data or code —e.g. participant privacy or use of data from a third party—those must be specified.

Reviewer #2: Yes

**********

PLOS authors have the option to publish the peer review history of their article (what does this mean?). If published, this will include your full peer review and any attached files.

If you choose “no”, your identity will remain anonymous but your review may still be made public.

**Do you want your identity to be public for this peer review?**  For information about this choice, including consent withdrawal, please see our Privacy Policy.

Reviewer #2: No

# Editor report: Acceptance letter

**DOI:** 10.1371/journal.pcbi.1012784.r008

**Authors:** Benjamin M. Althouse

PCOMPBIOL-D-25-00040R3

A fast numerical integration scheme for clonal expansion processes on graphs

Dear Dr Paterson,

I am pleased to inform you that your manuscript has been formally accepted for publication in PLOS Computational Biology. Your manuscript is now with our production department and you will be notified of the publication date in due course.

The corresponding author will soon be receiving a typeset proof for review, to ensure errors have not been introduced during production. Please review the PDF proof of your manuscript carefully, as this is the last chance to correct any errors. Please note that major changes, or those which affect the scientific understanding of the work, will likely cause delays to the publication date of your manuscript.

Soon after your final files are uploaded, unless you have opted out, the early version of your manuscript will be published online. The date of the early version will be your article's publication date. The final article will be published to the same URL, and all versions of the paper will be accessible to readers.

For Research, Software, and Methods articles, you will receive an invoice from PLOS for your publication fee after your manuscript has reached the completed accept phase. If you receive an email requesting payment before acceptance or for any other service, this may be a phishing scheme. Learn how to identify phishing emails and protect your accounts at https://explore.plos.org/phishing.

Thank you again for supporting PLOS Computational Biology and open-access publishing. We are looking forward to publishing your work!

With kind regards,

Sharmila Kamatchi

PLOS Computational Biology | Carlyle House, Carlyle Road, Cambridge CB4 3DN | United Kingdom ploscompbiol@plos.org | Phone +44 (0) 1223-442824 | ploscompbiol.org | @PLOSCompBiol
